# Nectar mimicry: a new phenomenon

**DOI:** 10.1038/s41598-020-63997-3

**Published:** 2020-04-27

**Authors:** Klaus Lunau, Zong-Xin Ren, Xiao-Qing Fan, Judith Trunschke, Graham H. Pyke, Hong Wang

**Affiliations:** 1 Institute of Sensory Ecology, Heinrich-Heine-University, Dusseldorf, Germany; 2 CAS Key Laboratory for Plant Diversity and Biogeography of East Asia, Kunming Institute of Botany, Chinese Academy of Sciences, Kunming, 650201 Kunming, P.R. China; 3 College of Life Sciences, Shaanxi Normal University, Xian, 710100 China; 4 Department of Biological Sciences, Macquarie University, Ryde, NSW 2019 Australia

**Keywords:** Ecology, Evolution

## Abstract

Nectar is the most common floral reward for flower-visiting flies, bees, bats and birds. Many flowers hide nectar in the floral tube and preclude sensing of nectar by flower-visitors from a distance. Even in those flowers that offer easily accessible nectar, the nectaries are mostly inconspicuous to the human eye and the amount of nectar is sparse. It is widely accepted that many flowers display nectar guides in order to direct flower-visitors towards the nectar. Using false colour photography, covering ultraviolet, blue and green ranges of wavelength, revealed a yet unknown conspicuousness of nectar, nectaries and false nectaries for bees due to concordant reflection in the ultraviolet range of wavelength. Nectars, many nectaries and false nectaries have glossy surfaces and reflect all incident light including UV-light. In most cases, this is not particularly conspicuous to the human eye, but highly visible for UV-sensitive insects, due to the fact that the glossy areas are often positioned in UV-absorbing central flower parts and thus produce a strong UV-signal. The optical contrast produced by the glossiness of small smooth areas in close proximity to nectar holders represents a widespread yet overlooked floral cue that nectarivorous flower-visitors might use to locate the floral nectar.

## Introduction

Nectar is the most common primary resource provided by flowers, but most nectar-feeding flower-visitors do not sense the nectar sugars and other ingredients of floral nectars until contact with their taste receptors^1^. At the same time, flower-visitors can use flower colours and odours as cues to indicate nectar-rewarding flowers. By contrast, direct sensing of nectar from a distance has only rarely been described^2–5^, possibly because most nectars are transparent liquids offered only in small amounts and often hidden in the flowers in order to be inconspicuous, or to prevent dilution by rain or evaporation due to heating.

However, nectar can sometimes be detected at a distance by flower-visitors^6^. The conspicuously coloured nectars of some flowers pollinated by birds^7^ and reptiles^8,9^ and the fluorescent nectars of some bee-pollinated flowers^10–12^ represent notable examples. In addition, the increased humidity in close proximity of the nectar is regarded as a reliable sensory cue for profitability assessment by nectar-foraging hawkmoths^13^. Also, olfactory cues of scented nectars may mediate detection of nectar-containing flowers by *Osmia* bees^14^.

The gloss of exposed nectar, sometimes referred to as glistening, shine or sheen, may represent another property, by which nectar could be remotely sensed^15,16^. For example, the staminodal nectaries of some *Pulsatilla* species produce glossy nectar^17^, and flies sometimes prefer to visit flowers with vividly glossy nectaries or pseudo-nectaries^18,19^. Already Sprengel^20^ found in 1793 not only that nectar droplets possess a smooth surface causing gloss, but also nectaries have a smooth surface^20^. For extrafloral nectaries, Pacini demonstrated that the nectar is not perceived as drops but as a glossy surface^21^.

However, some organs of flowers have been considered to act as false nectaries, resembling nectar but providing no such resource. Examples include the basal greenish and glossy floral guides of the Bittersweet *Solanum dulcamara* (Solanaceae)^22^, the glossy capitula of the staminodes of the Grass-of Parnassus *Parnassia palustris* (Celastraceae)^23^, the thickened glossy disc of *Cleome monophylla* (Cleomaceae)^24^, and the glossy pseudo-nectaries on the nectar leaves of *Nigella* species (Ranunculaceae)^25,26^. The glossy staminodes of *Parnassia palustris* have been regarded as false nectaries attracting flies; but experimental evidence is limited^18,23,27–29^. Despite the limited experimental evidence for visually detectable nectar and nectaries, some researchers take it for granted that nectar is glossy and thus nectar-seeking flower-visitors might detect nectar by means of this cue^24,30^.

In addition to genuine and false nectar, some secondary flower attributes such as overall flower colour and floral guides, which are often referred to as nectar guides, have been regarded as signals indicating nectar presence and/or location. Blue flower colour hue, for example, is associated with more nectar than other flower colour hues of bee-pollinated flowers^31^, and may thus act as a guide to nectar-rich flowers. Colour-changing floral guides seem to act as generally honest indicators of nectar presence in flowers, since the observed colour change of floral guides is correlated with changes in nectar production^32^.

Indicators of floral resources or its location (i.e. floral guides) represent a highly diverse phenomenon, and may appear as visual, acoustic, tactile or odourous guides. Visual floral guides, for example, may comprise black lines, dot guides of various colours, ultraviolet bull’s eyes, yellow and UV-absorbing pollen- and stamen-mimicking structures^33^. Some authors distinguish true and false nectar guides, depending on the presence or absence of nectar^34^.

Though nectar guides indicate the access to floral nectar and are guiding the approaching flower-visitor to a landing place or a place to insert its proboscis to reach the nectar^35^, nectar guides generally do not share any similarity with nectar or nectaries^24,36^, but in many cases mimic visual signals of pollen and anthers (i.e. the yellow and UV-absorbing colour^33,37^). For example, behavioural tests demonstrated that flower-naïve bumble bees, *Bombus terrestris*, innately respond to pollen-, or anther-mimicking floral guides by means of an antenna reaction, which guides them to the landing place^38^, but do not regularly handle these structures like real stamens with buzzing, mandible biting or other pollen-collecting behaviour^24,39^. Similarly, flower-naïve hoverflies, *Eristalis tenax*, extend their proboscis towards yellow and UV-absorbing pollen-mimicking nectar guides^40^, but accept both sugar water and pollen as a reward^41^.

Pollen- and anther-mimicking floral guides are rather uniform due to the yellow and UV-absorbing colour hue. However, Lunau *et al*. demonstrated that *Eristalis* hoverflies respond to this colour hue^42^, whereas bumble bees, respond to the higher colour saturation of the floral guide as compared to the rest of the corolla irrespective of the colour hue^38^.

In theory, it is possible that flowering plants independently evolve species-specific floral guides functioning as indicators for nectar resources, and the flower-visitors will learn the specific signals in each species’ nectar-indicating floral guide and preferably visit those flowers that display more conspicuous and honest signals. However, flowering plants are also likely to benefit from signal standardization or Mullerian mimicry^33^, when sharing common features of these floral guides with other species. Individual flower-visitors might find nectar more easily after switching to a new flower type if the new and the former type share floral signals that the flower-visitor has an innate or learnt preference to respond to. Such shared features of nectar and nectar-mimicking structures of floral guides, that could enhance the development of search images as well as reduce nectar robbing^43^ and handling time^44^, have not been demonstrated yet.

To demonstrate common features of nectar guides potentially suitable to guide naïve or experienced flower-visitors towards the nectar holder at close distance, a comparative study is needed in which suitable bee-visible floral signals are identified. The ideal sample would comprise all species of a highly diverse community of flowering plants as viewed by the eyes of the flower-visitors, and thus include the ultraviolet range of wavelength.

Based on previous findings, we investigated the properties of nectar, nectaries and nectar-mimicking structures, and explicitly tested two hypotheses. We firstly hypothesized that the gloss of openly displayed nectar is also present in floral guides in close proximity of nectar holders. Here, we define gloss as an optical property, caused by angle-dependent specular (mirror-like) reflections from smooth and flat surfaces^45^, that determines the visual appearance of objects. The factors that affect gloss are the coherence of light from the light source, the angle of incident light, the refractive index of the material and the topography of the surface^45^. The various stages of flowering (i.e. colour changes), movements of flower organs, and sun tracking were considered.

Since nectar guides are expected in close proximity of the nectar holder, the visibility in the ultraviolet range of wavelength might be crucial, since many center parts of flowers display a UV-absorbing colour^46^ providing an optimal background for ultraviolet signals. Therefore, we further hypothesized that these glossy signaling areas are more conspicuous in the ultraviolet range of wavelength, which is visible to most pollinators including bees, flies and butterflies, but not to humans.

The mountain ranges in the Southeastern part of the Himalayas provide a large diversity of flowering plants and pollinators including many species with easily accessible nectar^47–49^. We therefore carried out a survey of flower colours and floral colour patterns in the Hengduan mountains (Yunnan, China) using false colour photography in bee view^46^. We supported our findings by false colour photos taken beforehand in Germany, Switzerland and Australia. We aimed at documenting the floral colour pattern of as many species as possible, including even very small structures of flowers such as staminodes, protuberances, hairs, etc., which cannot be studied by a spectrophotometer due to their small size or rough surface. The objectives were as follows: identification of nectar-mimicking structures of flowers, documentation of inter-species similarity, description of features associated with glossy areas, and highlighting the unique features of this signal. All glossy areas of flowers and inflorescences located close to the center and thus potentially suited to guide flower-visitors towards the nectary are classified as nectar-mimicking floral guides.

## Material and methods

This study was carried out in the Yunnan Province in China in 2019. We located flowers on Yulong Snow Mountain at elevations ranging from 2700 m to 4200 m a.s.l. and either photographed them *in situ* or picked them in the field and transported them in plastic bags to the Lijiang Forest Ecosystem Research Station (at 3200 m a.s.l. on Yulong Snow Mountain, Yunnan Province, China), where they were photographed on the same or the next day. We also photographed flowers *in situ* on Baima Snow Mountain at 4300 m a.s.l, and in the Kunming Botanical Garden of the Kunming Institute of Botany, Chinese Academy of Sciences (Supplementary material, Tables S1–2). We complemented our observations with false colour photographs from our database taken in Germany, Switzerland and Western Australia in order to demonstrate better known examples and to demonstrate the wide distribution of the phenomenon. These false colour photos were made under similar camera settings as described for samples taken in China in 2017, in Germany and Switzerland in 2018, and in Western Australia in 2018.

High resolution false colour photography allows the determination of bee-visible colours of tiny floral structures and is suitable to register gloss^46^. Flower colours were measured by means of a modified Panasonic GH-1 camera. The low-pass filter of the camera has been removed in order to increase the sensitivity for ultraviolet light. The camera body was combined to an Ultra-Achromatic-Takumar 1:4.5/85 lens made of fused quartz that transmits UV-light. The photos were taken with the camera mounted to a tripod. Since the modified camera is sensitive to ultraviolet and infrared light, a UV-/IR-Cut filter transmitting light only between 400 nm and 700 nm was used to capture a normal reference colour picture. In addition, a UV-picture was captured from the identical position using a Baader UV-filter that transmits near ultraviolet light only. A white Teflon disc reflecting equal amounts of light in a range of wavelength from 300 nm to 700 nm was used for manual white balance before taking pictures. The aperture was set manually depending on light conditions and identical for the color photo and the UV-photo. The exposure time was set automatically. All pictures were taken outdoors without flash either with intact plants in the field or with clipped plants at the field station. The changes in ambient light conditions between taking the colour photo and the UV-photo were considered small that they are neglected here.

Using ImageJ (U.S. National Institutes of Health, Bethesda, Maryland, USA) both pictures were split into the RGB colour channels, and then a false colour photo was merged using the green channel of the colour picture as red, the blue channel of the colour picture as green, and the blue channel of the UV picture as blue channel for the merged picture. This way of merging has been demonstrated to produce the most informative false colour pictures^46^. The colour analysis was based on RGB code values of the false colour pictures. Due to the VIS- and UV-filter used and the ranges of wavelength covered by the RGB code the ultraviolet range of wavelength ranges from 300 nm to 400 nm, blue from 400 nm to 510 nm, and green from 450 nm to 640 nm. The colour code values refer to the RGB-values of the false colour photo; thus, UV refers to the blue channel of the UV-picture, blue refers to the green channel and green refers to the red channel of the false colour picture.

Species, from which photos in good quality could be taken in sunshine were selected for quantitative analysis of glossy structures (Supplementary material, Tables S3–5). The difference in the reflection in the ultraviolet, blue, and green ranges of wavelengths was quantified using IrfanView image’s histogram (free from www.irfanview.com). A uniform non-decomposed area of the glossy target area on the false color picture was selected. The average intensity for the red, green and blue channel of the false color photos with RGB code values between 0 and 255 was used for color evaluation using IrfanView image’s histogram. For comparison, an adjacent, non-glossy, and uniformly coloured area of the same structure was measured in the same way. In all cases the adjacent area had the same human-visible colour and UV-reflection property as the glossy target area as far as it could be judged from the human eye and photos.

The statistical analysis compares the difference in reflection between the glossy area and the adjacent non-glossy area for the three ranges of wavelength (Supplementary material, Figs. S1–2). The values for the difference in reflection between the glossy area and the adjacent non-glossy area of each range of wavelength were checked for normal distribution using a Shapiro-Wilk test. Since the data were not normally distributed a paired, two-sided Wilcoxon Signed Rank Test was performed.

## Results

**Figure 1:**
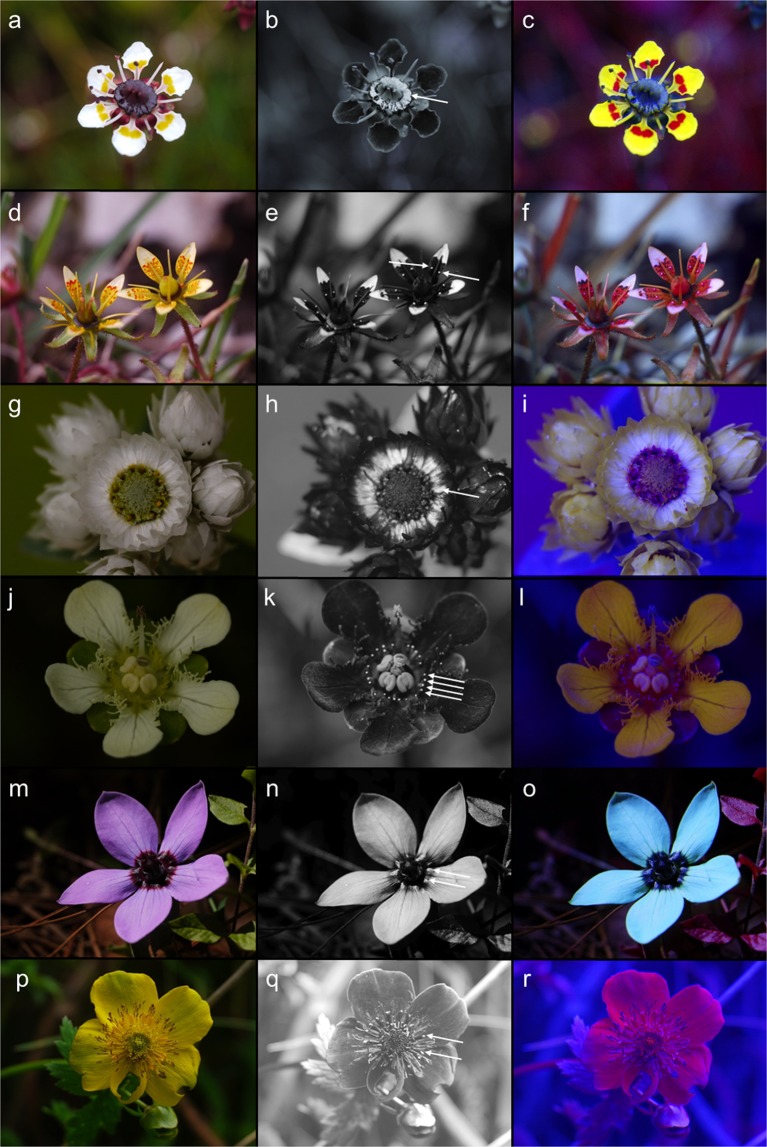
Colour photo (left), UV-photo (middle), and false colour photo in bee view (right) of (**a**–**c**) disc nectary in *Saxifraga melanocentra*, (**d**–**f**) two glossy protuberances per petal in *S. signata*, (**g**–**i**) ring of glossy basal parts of petals in *Anaphalis nepalensis*, (**j**–**l**) glossy tips of staminodes in *Parnassia wightiana*, (**m**–**o**) dark, glossy petal base in *Codonopsis graminifolia*, and (**p**–**r**) glossy tips staminodes in *Trollius yunnanensis*. Arrows in the UV-photos indicate glossy areas. The photos were taken on Yulong Snow Mountain and Baima Snow Mountain in China (Yunnan).

**Figure 2:**
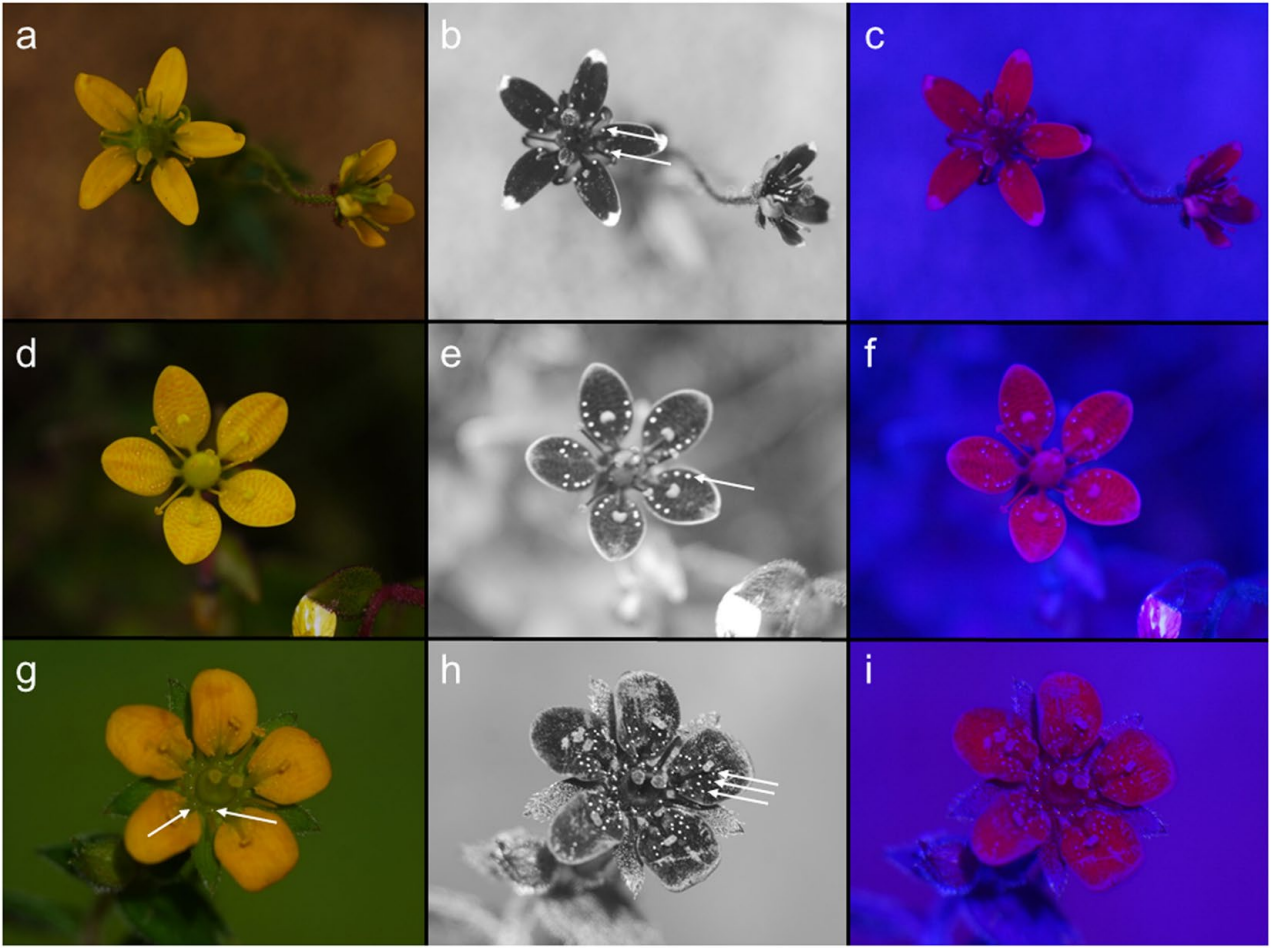
Colour photo (left), UV-photo (middle), and false colour photo in bee view (right) of glossy nectaries and protuberances on the callose petals of (**a**–**c**) *Saxifraga unguiculata*, (**d**–**f**) *S. diversifolia*, and (**g**–**i**) *S. nigroglandulosa*. Arrows in the UV-photos indicate glossy areas; arrows in g) indicate glossy nectar. The photos were taken on Yulong Snow Mountain and Baima Snow Mountain in China (Yunnan).

**Figure 3:**
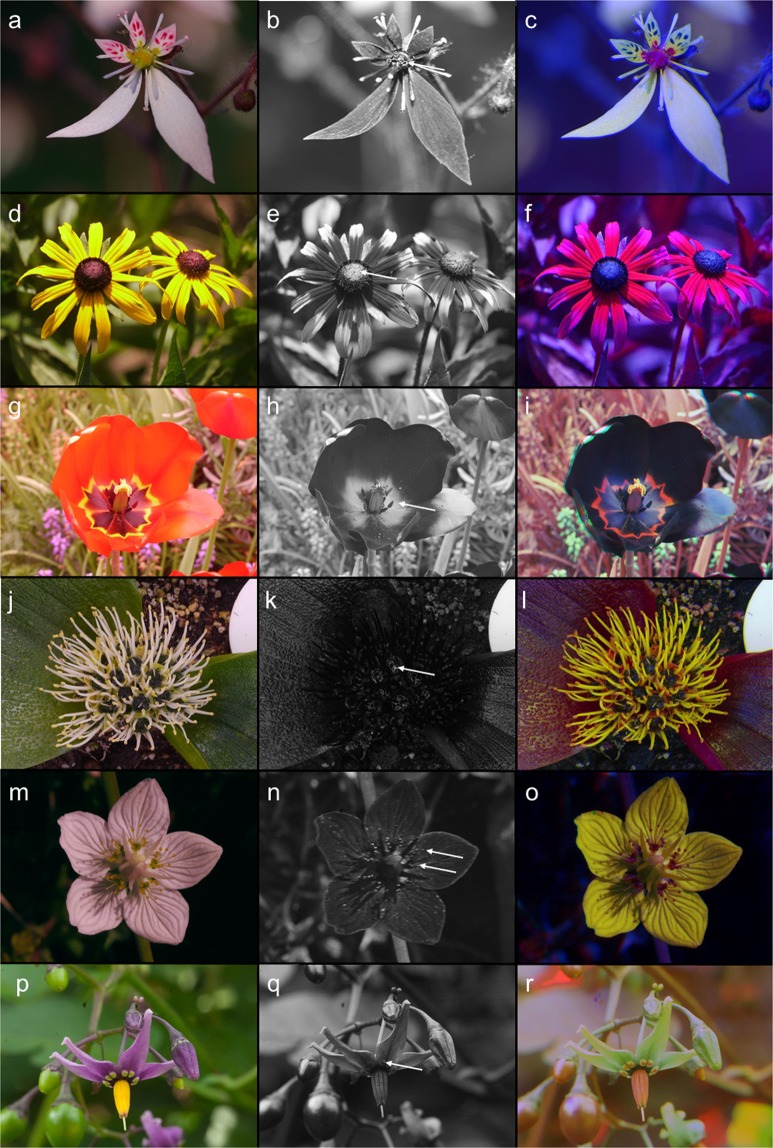
Colour photo (left), UV-photo (middle), and false colour photo in bee view (right) of (**a**–**c**) glossy nectary disc in *Saxifraga ferruginea*, (**d**–**f**) dark glossy anthers of disc florets in a garden form of *Rudbeckia fuliginosa*, (**g**–**i**) dark glossy base of petals in a garden form of *Tulipa gesneriana*, (**j**–**l**) glossy dark nectar in *Massonia pustulata*, (**m**–**o**) glossy staminodes in *Parnassia palustris*, and (**p**–**r**) false nectaries on the petals of *Solanum dulcamara*. Arrows in the UV-photos indicate glossy areas or glossy nectar. The photos were taken in Germany or Switzerland.

We found that flowers and inflorescences of many plant species had glossy areas that exhibited a striking visual inter-species similarity, with glossy parts in close proximity to the floral center (Figs. 1–3). The colour, false colour, and UV-photos of the non-homologous glossy areas of flowers document a common property, which is based on the total reflection of light (i.e. gloss), in a UV-absorbing direct surrounding causing highest conspicuousness in the UV, and visibility to bees (Figs. 1–3).

We also found that such glossy areas near the floral center were common amongst plant species with radially symmetrical flowers and rare for species with zygomorphic flowers. 309 species of flowering plants in the Himalayas were photographed and identified, out of these 174 species (56%) had radially symmetrical flowers. Out of the 174 species with radially symmetrical flowers, 23 had no nectar, 88 hidden nectar, and 63 potentially visible or visible nectar (personal observation; Supplementary material, Tables S1–2). Glossy areas on flowers/inflorescences near the floral center and hence potentially serving as nectar guides were found in 38 species with radially symmetrical flowers and in 1 zygomorphic species (*Saxifraga rufescens*; Supplementary material, Tables S1–2).

We also found that many plants exhibited apparent pollen mimicry, with pollen and nectar mimicry overlapping. Pollen-mimicking yellow and UV-absorbing floral guides as described by Lunau^36,37^ were found in 75 species. Both glossy structures, potentially mimicking nectar, and pollen-mimicking structures were found in 11 species, and, in an additional 6 species, the bee-yellow and UV-absorbing staminodes were categorized as both stamen mimics as well as glossy areas, potentially mimicking nectar (Supplementary material, Table S1).

There is an obvious similarity between these glossy areas and nectar per se, since both reflect all incident light and are thus visible on the colour photo, the UV-photo, and the false colour picture. The glossiness of nectar is caused by total reflection of light that is visible for bees and flies, which are UV-sensitive and red-insensitive, as demonstrated by UV-photos and false colour photos independent of nectar presentation (i.e., whether the nectar is offered as large-scale secretion as in *Saxifraga melanocentra* - Fig. 1a–c, or as droplets as in *Saxifraga signata*; personal observation - Fig. 1d–f). Similar results are found in flowers which either offer transparent nectar like *Saxifraga stolonifera* (Fig. 3a–c) or coloured nectar like *Massonia pustulata* (Asparagaceae) (personal observation; Fig. 3j–l).

The glossy areas are independent of the underlying colour hue. Contrast in the UV was present in all species studied, since the glossy areas were located in the UV-absorbing center parts of the flowers. In some yellow-flowered *Saxifraga* species (Saxifragaceae) the colour hue of the glossy protuberances of the petals are yellow (Figs. 1, 2). In *Saxifraga melanocentra* the colour hue of the floral disc is dark purple (Fig. 1a–c). The staminodes of *Trollius yunnanensis* (Ranunculaceae) are orange (Fig. 1p–r); several *Parnassia* species (Celastraceae) possess green or yellow staminodes (Figs. 1j–l, 3m–o). In *Codonopsis graminifolia* (Campanulaceae) the basal glossy part of the petals is black (Fig. 1m–o). In *Anaphalis nepalensis* (Asteraceae) the petals are white and glossy parts are apparent only if the flowers are fully opened (Fig. 1g–i).

The yellow-flowering *Saxifraga* species from the Yulong Snow Mountain exhibit variation in the number of glossy areas (Fig. 2). The number of glossy protuberances varies between 2 per petal in *S. unguiculata* (Fig. 2a–c), up to10 per petal in *S. diversifolia* (Fig. 2d–f) and more than 12 per petal in *S. nigroglandulosa* (Fig. 2g–i), but the visual appearance of the glossy protuberances is rather similar. In the photo of *S. nigroglandulosa* the gloss produced by real nectar droplets and by the nectar-mimicking protuberances on the petals can be directly compared (Fig. 2).

Despite the differences in colour hue for the human eye, the appearance of the glossy areas on UV-pictures is rather uniform, in that they reflect ultraviolet light if they have been photographed under appropriate light conditions (Figs. 1, 2). The gloss comes as a total reflection of light and thus appears in all ranges of wavelength. This holds also for the glossy areas in the floral center, like the black anthers of *Rudbeckia fuliginosa* (Asteraceae, Fig. 3d–f), the black basal parts of the petals of *Tulipa gesneriana* (Liliaceae, Fig. 3g–i), the yellow staminodes of *Parnassia palustris* (Fig. 3m–o), and the green floral guides of the petals of *Solanum dulcamara* (Solanaceae, Fig. 3p–r).

Since nectar is offered in the center of flowers, which are mostly UV-absorbing, the gloss is visible particularly in the ultraviolet range of wavelength, but less in those ranges of wavelength that are reflected in the central flower parts and represented by the colour picture, e.g. blue in blue and purple flowers, green in yellow flowers and blue and green in white flowers.

**Figure 4:**
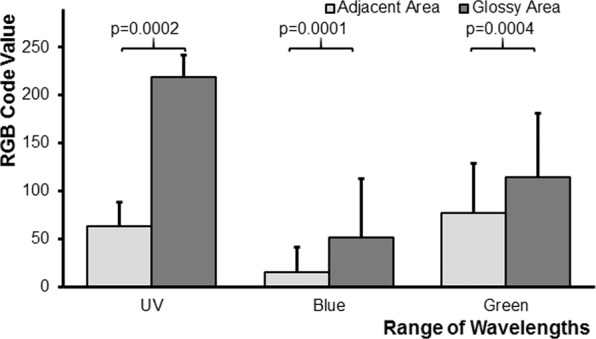
Mean RGB colour code values and SEM (standard error of the mean) for nectar indicating structures of flowers from the Himalayas (*Parnassia wightiana, P. delavayi, P. yunnanensis*, *Saxifraga unguiculata, S. nigroglandulosa, S. diversifolia, S. melanocentra, Codonopsis graminifolia, S. signata, Anaphalis nepalensis, Trollius yunnanensis, Potentilla lancinata* cf; see Supplementary material, Table S3). The values for the glossy area are shown in dark gray, those for the adjacent non-glossy area in light gray. P-values indicate significant differences between the reflection of the glossy area and the adjacent non-glossy area for the ranges of wavelength according to a paired, two-tailed Wilcoxon Signed Rank Test.

**Figure 5:**
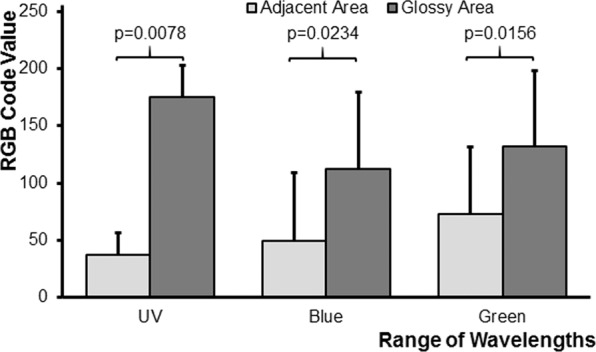
Mean RGB colour code values and SEM (standard error of the mean) for nectar indicating structures of flower from Germany, Switzerland, and Western Australia (*Saxifraga stolonifera*, *Rudbeckia fuliginosa, Tulipa gesneriana, Massonia pustulata, Parnassia palustris, Solanum dulcamara, Anemone coronaria, Swainsonia formosa*; see Supplementary material, Table S4). The values for the glossy area are shown in dark gray, those for the adjacent non-glossy area in light gray. P-values indicate significant differences between the reflection of the glossy area and the adjacent non-glossy area for the ranges of wavelength according to a paired, two-tailed Wilcoxon Signed Rank Test.

The glossy area of the measured floral organ is brighter in all ranges of wavelength as compared to the adjacent non-gloss area of the same structure (Figs. 4, 5). In most cases, the glossy area and the directly adjacent non-glossy area of each flower, measured for comparison, had the same pigment-based colour to the human and to the bee eye (Supplementary material, Figs. S1–2, Table S5). The reflection of the glossy area is significantly higher in all ranges of wavelength than that of the adjacent area; however, the difference in the reflection between the glossy area and the respective non-glossy same-coloured adjacent area is most pronounced in the ultraviolet range of wavelength (Figs. 4, 5). In all cases, the glossy area and the adjacent non-glossy area belong to the same floral organ with a smooth surface. The gloss is thus caused by the mirror-angle in which the photo was taken, whereas the adjacent areas were not photographed in the mirror angle and thus not glossy. The smoothness of the glossy and adjacent areas was checked under a microscope, but not documented by measurements with a glossmeter or by microphotography.

## Discussion

The photographic survey of floral colour pattern in the Yulong and Baima Snow Mountains, based on false colour photos in bee view and on colour analysis, revealed glossy areas on various flower parts of many species. The gloss is probably caused by the smoothness of the epidermal surface and visible in the mirror angle. Due to the inter-species similarity of these glossy areas, a new type of floral guide is described with gloss and close proximity to the nectar holder as most important attributes. The gloss is restricted to small areas mostly because the overall organs are small, but also because the smooth areas are convex or concave and thus produce gloss only in a distinct angle of viewing that is identical to the angle of incidence, i.e. the mirror angle.

The visual cues associated with these glossy areas include total reflection of light resulting in a human-white and bee-white colour hue that produces colour contrast and brightness contrast to its immediate surrounding located at non-glossy parts of the same structure. It has been suggested that the glossy areas are mimicking features of real nectar^50,51^. Indeed, openly offered floral nectar displays a visual cue to flower-visitors with gloss as a main property if appropriately exposed to light. The similarity between real nectar and glossy nectar guides warrants categorizing these glossy nectar guides as nectar mimicry^46^.

The gloss can be associated with any flower colour hue; the resulting signal, however, is similar due to total reflection of incident light and thus independent of the underlying pigment colour and displaying a sparkle of a white and UV-reflecting colour. Despite this diversity of underlying colour hues for nectar-mimicking structures, many structures are UV-absorbing, and thus display a stronger contrast in the ultraviolet range of wavelength between the glossy and the non-glossy adjacent area of the same organ, which might increase its conspicuousness to UV-sensitive bees. The absorption of ultraviolet light in the center part of flowers is known to protect vulnerable floral organs against ultraviolet light reflected from the petals^52^. Since the gloss is restricted to a small area and the direction of the reflected light steadily moves due to the position of the sun, the glossy floral guides do not seem to provide protection against reflected ultraviolet light. In addition, dark or black glossy structures like in *Codonospsis graminifolia* and *Saxifraga melanocentra* produce a colour contrast between the glossy area and the non-glossy surrounding area covering all ranges of wavelength and thus the largest possible brightness contrast. It is remarkable that also real nectaries differ in their underlying colour, although most nectaries seem to be associated with a yellow colour hue^24,53^. It is known that gloss effects on flowers are found in combination of all colours, but are most conspicuous in the ultraviolet range of wavelength^54–56^.

The findings of this study suggest that the glossy areas are relatively small as compared to the entire flower/inflorescence and rather uniform among species, thus bees might use them for locating nectar rather than for flower discrimination. Laboratory studies indicate that bees can perceive the structural colours caused by nanostructures, if presented as the sole variable cue on relatively large-sized artificial flowers^57^, but evidence from field experiments is still missing^58^. Whitney *et al*. argue that the effects of structural colours should be small in order not to perturb colour discrimination^57^. The results of this study suggest a function of glossy areas on flowers, i.e. locating the nectar source, in which colour discrimination plays no role.

There is much evidence that flower-visitors respond to visual floral guides^32,33,36–41^; there is also some evidence that visual cues of glossy structures might guide flower-visitors towards the nectar. In behavioural tests, naïve as well as experienced hoverflies, *Tubifera pendula* and *Syrphus balteatus*, did not respond to the clipping of the staminodes of *Parnassia palustris* as would be expected if they are nectar guides^27,28,59^. Contrary, Kugler found that inexperienced *Lucilia* flies spontaneously approach glossy drop-shaped objects and interpreted this preference as an innate search image for nectar^29^. These behavioural studies suggest that at least some flies might exhibit an innate response towards glossy areas.

The glossy areas of flowers might indicate a nectar-mimicking structure that either acts as true nectar guide and thus guides flower-visitors towards the nectar on the flower, or, alternatively, as false nectar guide by means of manipulating (sensu Pyke^60^) the flower-visitors’ behaviour in nectarless flowers.

Glossy areas on flowers imply distinct smooth surface properties. Conical epidermis cells are common on adaxial surfaces of petals in angiosperms^61^ featuring our finding of glossy areas on flowers. The fact that smooth epidermal cells in the most basal parts of the adaxial side of petals might serve as a tongue guide and tongue channel towards the nectar (sensu Erickson & Garment^62^), by which the tongue slips easier on a smooth and inclined surface towards the nectar, might point to a previous or an additional function of glossy areas on flowers.

Black central areas of flowers have been described in a totally different context, namely these areas serve as rendezvous-sites for dipterans, hymenopterans, and coleopterans; they also seem to possess glossy structures, which in this case mimic properties of the pollinators, mostly the glossy wings. Examples are the floral guides of *Gorteria* flowers pollinated by beeflies, and *Papaver rhoeas* and other flowers pollinated by scarabaeid beetles, and sexually deceptive orchids^63–66^. Remarkably, in some cases, these black structures do not exhibit gloss, but mimic the sparkling total reflection of light by means of white and UV-reflecting spots within or adjacent to the black area^67–69^. The uncertainty to categorize such glossy structures to nectar mimicry or pollinator mimicry without experimental data is apparent for example from observations on the pollination of *Pelargonium tricolor* a flower with a shiny embossed dark central structure^70^.

Spectral reflection data of glossy areas have never been reported, probably because the areas are too small and because recordings of the spectral reflection of flowers are not taken in the mirror angle and thus do not register gloss. High resolution photography in the VIS and UV ranges of wavelength and subsequent renders enable the colour analysis of tiny structures on flowers.

The existence of glossy floral guides, indicating nectar location, has often been reported^24^. For example, the glossy staminodes of *Parnassia palustris*, glistening hairs on *Erodium* petals, and the floral guides of *Solanum dulcamara* have been described as false nectaries^5,22,23^. However, the terms pseudo-nectary, false nectary, nectar mimicry, and nectar guide seem to be poorly defined in pollination ecology textbooks^71–73^. Even Johnson & Schiestl in a textbook about floral mimicry^74^ neither mention pseudo-nectaries nor false nectaries; they discuss, however, that honest signaling of nectar availability might depend on nectar scents rather than visual cues^75,76^, since most flowers hide their nectar. Also, the review of Parachnowitsch *et al*. about the evolutionary ecology of nectar does not mention visual properties of nectar or nectaries like gloss^77^. By contrast, some researchers carefully distinguish between false and true nectar guides, as in the case of false nectar guides of the Australian nectarless *Diuris* orchids^78^. Since evidence how these nectar guides work is lacking^58^, many researchers might hesitate to state that deception or mimicry is involved. Further research is needed to identify the locations of these signals and to clarify if learning is necessary to respond or not.

Our findings also suggest that the phenomenon of glossy structures as true nectar guides is more widespread than previously thought, since it was not only found in open flowers in the Himalayas, but also in Germany and Switzerland. In addition, examples from other continents, i.e. *Swainsonia formosa* from Australia and *Massonia pustulata* from Africa were added. We conclude that our study provides evidence for the presence of a new, yet undescribed type of floral guide that mimics true nectar and thus can be categorized as nectar guide. Our study encourages the re-evaluation of floral colour patterns with the possibility of recording gloss particularly of smooth-surfaced small flower parts of open flowers. Our findings also encourage follow-up studies to investigate behavioural responses of bees and flies to small glossy areas of flowers in order to demonstrate their function as nectar-guides. The response to these nectar guides must not necessarily be innate, since many flowers with open nectaries display glossy nectar and flower-visitors can easily learn this cue through conditioning. Despite their small size, the gloss of these structures might also be used by nectar-feeding insects as a visual cue for finding access to nectar even before touching the flowers, since the gloss is only visible in a distinct viewing angle and thus might pop up to a flower-visitor when approaching a flower and is thus easily detectable.

## Supplementary information

Supplementary Information.
