# Supplementary material for: Nectar mimicry: a new phenomenon
Source: Sci Rep. 2020 Apr 27;10:7039. doi: 10.1038/s41598-020-63997-3 (PMC7184725; doi:10.1038/s41598-020-63997-3)
Supplement: Supplementary file 1 — Supplementary Information. [file 41598_2020_63997_MOESM1_ESM.pdf]

Nectar mimicry: a new phenomenon

Klaus Lunau[1,2], Zong-Xin Ren[2], Xiao-Qing Fan[2,3], Judith Trunschke[2], Graham H. Pyke[2,4], & Hong Wang[2]

[1]Institute of Sensory Ecology, Heinrich-Heine-University, Dusseldorf, Germany

[2]CAS Key Laboratory for Plant Diversity and Biogeography of East Asia, Kunming Institute of Botany, Chinese Academy of Sciences, Kunming 650201 Kunming, PR China

[3]College of Life Sciences, Shaanxi Normal University, Xian 710100, China

[4]Dept of Biological Sciences, Macquarie University, Ryde, NSW 2019, Australia

Corresponding authors: Klaus Lunau, E-mail: Klaus.Lunau@hhu.de
Zong-Xin Ren, E-mail: renzongxin@mail.kib.ac.cn

Table S1. Species list containing identified 309 species of the Yulong and Baima Snow Mountains and categorized features of nectar, pollen, glossy structures, and altitude.

FAMILY	SPECIES	FlORAL SYMMETRY	NECTAR	GLOSSY STRUCTURE	INVISIBLE POLLEN & POLLEN MIMICRY	ALITUDE
Solanaceae	Nicandra physalodes	radially symmetrical flower	no nectar	glossy basal part of petal	floral guide mimics anther colour	2700
Campanulaceae	Codonopsis bulleyana	radially symmetrical flower	visible	glossy basal part of petal		3900
Campanulaceae	Codonopsis convolvulacea	radially symmetrical flower	visible	glossy basal part of petal		3200
Campanulaceae	Codonopsis graminifolia	radially symmetrical flower	visible	glossy basal part of petal		3200
Erodiaceae	Geranium delavayi	radially symmetrical flower	visible	glossy basal part of petal		3200
Erodiaceae	Geranium strictipes	radially symmetrical flower	visible	glossy basal part of petal		3200
Gentianaceae	Swertia cincta	radially symmetrical flower	visible	glossy basal part of petal		3200
Gentianaceae	Swertia macrosperma	radially symmetrical flower	visible	glossy basal part of petal	floral guide mimics anther colour	3200
Gentianaceae	Swertia punicea	radially symmetrical flower	visible	glossy basal part of petal		3200 (Yunshanping)
Rosaceae	Potentilla anserina	radially symmetrical flower	visible	glossy central structures	floral guide and stigmata mimic anther colour	3200
Rosaceae	Potentilla articulata	radially symmetrical flower	visible	glossy central structures	floral guide mimics anther colour	4200
Rosaceae	Potentilla coriandrifolia	radially symmetrical flower	visible	glossy central structures	floral guide mimics anther colour	4200
Rosaceae	Potentilla eriocarpa	radially symmetrical flower	visible	glossy central structures	floral guide mimics anther colour	3600
Rosaceae	Potentilla fruticosa	radially symmetrical flower	visible	glossy central structures		3600
Rosaceae	Potentilla glabra	radially symmetrical flower	visible	glossy central structures		3600
Rosaceae	Potentilla lancinata	radially symmetrical flower	visible	glossy central structures	stigma mimics stamen	2700
Rosaceae	Potentilla peduncularis var. curta	radially symmetrical flower	visible	glossy central structures	floral guide mimics anther colour	3600
Saxifragaceae	Saxifraga melanocentra	radially symmetrical flower	visible	glossy disc	floral guide mimics anther colour	4100
Saxifragaceae	Saxifraga rufescens	zygomorphic flower	visible	glossy disc	flower center mimics stamen colour	3400
Campanulaceae	Cyananthus formosus	radially symmetrical flower	hidden	glossy floral guide		4100
Campanulaceae	Cyananthus longiflorus	radially symmetrical flower	hidden	glossy floral guide		3200
Campanulaceae	Cyanathus hookeri	radially symmetrical flower	hidden	glossy floral guide		3400
Asteraceae	Anaphalis chlamydophylla	radially symmetrical inflorescence	hidden	glossy floral guide		4100
Asteraceae	Anaphalis nepalensis	radially symmetrical inflorescence	hidden	glossy floral guide		3200
Asteraceae	Anaphalis yunnanensis cf	radially symmetrical inflorescence	hidden	glossy floral guide	anthers and pollen visible	3200

Saxifragaceae	Saxifraga aristulata	radially symmetrical flower	visible	glossy protuberances		4200
Saxifragaceae	Saxifraga diversifolia,	radially symmetrical flower	visible	glossy protuberances		3200
Saxifragaceae	Saxifraga filicaulis	radially symmetrical flower	visible	glossy protuberances		3200
Saxifragaceae	Saxifraga gemmipara	radially symmetrical flower	visible	glossy protuberances	floral guide mimics anther colour	3200 (Yunshanping)
Saxifragaceae	Saxifraga hispidula	radially symmetrical flower	visible	glossy protuberances		4200
Saxifragaceae	Saxifraga nigroglandulosa	radially symmetrical flower	visible	glossy protuberances		4200
Saxifragaceae	Saxifraga strigosa	radially symmetrical flower	visible	glossy protuberances		3400
Saxifragaceae	Saxifraga wallichiana	radially symmetrical flower	visible	glossy protuberances		4200
Ranunculaceae	Trollius yunnanensis	radially symmetrical flower	no nectar	glossy staminodes	staminodes mimic stamens	3200
Celastraceae	Parnassia delavayi	radially symmetrical flower	visible	glossy staminodes	staminodes mimic stamens	3200
Celastraceae	Parnassia mysorensis	radially symmetrical flower	visible	glossy staminodes	staminodes mimic stamens	3200
Celastraceae	Parnassia tenella	radially symmetrical flower	visible	glossy staminodes	staminodes mimic stamens	3200
Celastraceae	Parnassia wightiana	radially symmetrical flower	visible	glossy staminodes	staminodes mimic stamens	2700
Celastraceae	Parnassia yunnanensis	radially symmetrical flower	visible	glossy staminodes	staminodes mimic stamens	4100
Boraginaceae	Cynoglossum amabile	radially symmetrical flower	hidden			3200
Boraginaceae	Microula forrestii	radially symmetrical flower	hidden		floral guide mimics anther colour	4100
Boraginaceae	Onosma confertum	radially symmetrical flower	hidden			3200
Brassicaceae	Megacarpaea delavayi	radially symmetrical flower	hidden			3200
Brassicaceae	Nasturtium officinale	radially symmetrical flower	hidden			4100
Brassicaceae	Rorippa elata	radially symmetrical flower	hidden			3400
Campanulaceae	Adenophora capillaris subsp.leptosepala	radially symmetrical flower	hidden			3200
Campanulaceae	Adenophora coelestis	radially symmetrical flower	hidden			3200
Campanulaceae	Campanula crenulata	radially symmetrical flower	hidden			3900
Campanulaceae	Campanula pallida	radially symmetrical flower	hidden			3200
Campanulaceae	Cyananthus inflatus	radially symmetrical flower	hidden			3600
Campanulaceae	Cyananthus macrocalyx	radially symmetrical flower	hidden		floral guide mimics anther colour	3200
Caprifoliaceae	Morina nepalensis var. alba	radially symmetrical flower	hidden			3600
Caprifoliaceae	Morina nepalensis var.delavayi	radially symmetrical flower	hidden			3200
Daphnaceae	Stellera chamaejasme	radially symmetrical flower	hidden			3600
Dianthaceae	Silene asclepiadea	radially symmetrical flower	hidden			3400
Dianthaceae	Silene cardiopetala	radially symmetrical flower	hidden			3200

Dianthaceae	Silene melanantha	radially symmetrical flower	hidden			3600
Ericaceae	Monotropa hypopitys	radially symmetrical flower	hidden			3200
Ericaceae	Pieris formosa	radially symmetrical flower	hidden			3400
Ericaceae	Rhododendron primulaeflorum	radially symmetrical flower	hidden			3200
Ericaceae	Vaccinium fragile	radially symmetrical flower	hidden			3200
Gentianaceae	Comastoma cyananthiflorum var. cyananthiflorum	radially symmetrical flower	hidden			4100
Gentianaceae	Gentiana crassicaulis	radially symmetrical flower	hidden			3200
Gentianaceae	Gentiana serra	radially symmetrical flower	hidden			3200 (Yunshanping)
Gentianaceae	Gentiana sinoornata	radially symmetrical flower	hidden			4100
Gentianaceae	Gentiana yunnanensis	radially symmetrical flower	hidden			3200
Gentianaceae	Gentianopsis grandis	radially symmetrical flower	hidden			2700
Gentianaceae	Halenia elliptica	radially symmetrical flower	hidden			3200
Gentianaceae	Lomatogonium lijiangense	radially symmetrical flower	hidden			4200
Gesneriaceae	Corallodiscus lanuginosus	radially symmetrical flower	hidden			3200
Gesneriaceae	Oreocharis forrestii	radially symmetrical flower	hidden			3200
Liliaceae	Disporum brachystemon	radially symmetrical flower	hidden			3200
Oleaceae	Jasminum officinale	radially symmetrical flower	hidden			2700
Oleaceae	Jasminum humile	radially symmetrical flower	hidden		floral guide mimics anther colour	3200
Onagraceae	Epilobium royleanum	radially symmetrical flower	hidden		stigmata mimic stamens	4100
Primulacae	Primula sikkimensis cf	radially symmetrical flower	hidden			3200
Primulaceae	Androsace spinulifera	radially symmetrical flower	hidden		floral guide mimics anther colour	3200
Primulaceae	Lysimachia christiniae	radially symmetrical flower	hidden			3200
Primulaceae	Lysimachia pumila	radially symmetrical flower	hidden			3200
Primulaceae	Primula beesiana	radially symmetrical flower	hidden		floral guide mimics anther colour	3400
Primulaceae	Primula bella	radially symmetrical flower	hidden			4100
Primulaceae	Primula bulleyana	radially symmetrical flower	hidden		floral guide mimics anther colour	3200
Primulaceae	Primula poisonii	radially symmetrical flower	hidden		floral guide mimics anther colour	3200
Primulaceae	Primula poissonii	radially symmetrical flower	hidden		floral guide mimics anther colour	2700
Primulaceae	Primula vialii	radially symmetrical flower	hidden			3200
Primulaceae	Primula yunnanensis	radially symmetrical flower	hidden			3200
Rosaceae	Neillia gracilis	radially symmetrical flower	hidden			3400

Rubiaceae	Leptodermis potanini	radially symmetrical flower	hidden			2700
Scrophulariaceae	Buddleja fallowiana	radially symmetrical flower	hidden			2700
Commelinaceae	Cyanotis vaga	radially symmetrical flower	no nectar		staminodes mimic stamens	2700
Hypoxidaceae	Hypoxis aurea	radially symmetrical flower	no nectar			2700
Papaveraceae	Meconopis delavayi	radially symmetrical flower	no nectar			4100
Papaveraceae	Meconopis rudis	radially symmetrical flower	no nectar			4100
Plantaginaceae	Plantago asiatica subsp.erosa	radially symmetrical flower	no nectar			3400
Ranuculaceae	Anemone rivularis var. flore-minore	radially symmetrical flower	no nectar		floral guide mimics anther colour	3400
Ranuculaceae	Anemone trullifolia var.holophylla	radially symmetrical flower	no nectar			3600
Ranuculaceae	Ranunculus yunnanensis	radially symmetrical flower	no nectar			3600
Ranuculaceae	Thalictrum javanicum	radially symmetrical flower	no nectar			3200
Ranunculaceae	Anemone hupehensis	radially symmetrical flower	no nectar			3200
Ranunculaceae	Anemone rupicola	radially symmetrical flower	no nectar			4100
Ranunculaceae	Caltha palustris	radially symmetrical flower	no nectar		floral guide mimics anther colour	3400
Ranunculaceae	Caltha scaposa	radially symmetrical flower	no nectar			4200 (Baima)
Ranunculaceae	Clematis chrysocoma	radially symmetrical flower	no nectar		stigmata mimic stamens	3200
Ranunculaceae	Clematis peterae	radially symmetrical flower	no nectar			2700
Ranunculaceae	Clematis ranunculoides	radially symmetrical flower	no nectar			3200
Ranunculaceae	Ranunculus nephenogenes	radially symmetrical flower	no nectar		floral guide mimics anther colour	3200
Ranunculaceae	Thalictrum delavayi	radially symmetrical flower	no nectar			2700
Ranunculaceae	Anemone rivularis	radially symmetrical flower	no nectar			3200
Solanaceae	Anisodus acutangulus	radially symmetrical flower	no nectar			3200
Crassulaceae	Rhodiola chrysanthemifolia	radially symmetrical flower	potentially visible			4100
Crassulaceae	Sedum trullipetalum	radially symmetrical flower	potentially visible			3200
Dianthaceae	Stellaria vestita	radially symmetrical flower	potentially visible			3200
Dianthaceae	Stellaria yunnanensis	radially symmetrical flower	potentially visible			3400
Hypericaceae	Hypericum chosiyanum	radially symmetrical flower	potentially visible			2700
Polygonaceae	Fagopyrum dibotrys	radially symmetrical flower	potentially visible			3200
Polygonaceae	Polygonum nepalense	radially symmetrical flower	potentially visible			2700

Polygonaceae	Polygonum paleaceum	radially symmetrical flower	potentially visible			3400
Polygonaceae	Polygonum viviparum	radially symmetrical flower	potentially visible			4100
Polygonaceaea	Polygonum macrophyllum	radially symmetrical flower	potentially visible			3600
Saxifragacae	Saxifraga pallida	radially symmetrical flower	potentially visible		floral guide mimics anther colour	3400
Tofieldiaceae	Tofieldia divergens	radially symmetrical flower	potentially visible			2700
Amaryllidaceae	Allium beesianum	radially symmetrical flower	visible			4200
Amaryllidaceae	Allium mairei	radially symmetrical flower	visible			3900
Apiaceae	Bupleurum rockii	radially symmetrical flower	visible			3200
Apiaceae	Heracleum yungningense	radially symmetrical flower	visible			3200
Apiaceae	Ligusticum pteridophyllum	radially symmetrical flower	visible			3200
Apiaceae	Ligusticum sinense Oliv	radially symmetrical flower	visible			3200
Apiaceae	Pleurospermum foetens	radially symmetrical flower	visible			4100
Asparagaceae	Ophiopogon bodinieri	radially symmetrical flower	visible			2700
Balanophoraceae	Balanophora involucrata	radially symmetrical flower	visible			3200
Berberidaceae	Berberis pruinosa	radially symmetrical flower	visible			3200
Brassicaceae	Arabis paniculata	radially symmetrical flower	visible			3200
Brassicaceae	Cardamine gracilis	radially symmetrical flower	visible			3200
Brassicaceae	Cardamine granulifera	radially symmetrical flower	visible			3200
Brassicaceae	Dipoma iberideum	radially symmetrical flower	visible			4100
Caprifoliaceae	Dipsacus asper	radially symmetrical flower	visible			3200
Caprifoliaceae	Nardostachys jatamansi	radially symmetrical flower	visible			3200
Caprifoliaceae	Pterocephalus hookeri	radially symmetrical flower	visible			3200
Caprifoliaceae	Valeriana hardwickii	radially symmetrical flower	visible			3200
Dianthaceae	Arenaria barbata	radially symmetrical flower	visible			3400
Dianthaceae	Arenaria leptophylla	radially symmetrical flower	visible			4100
Rutaceae	Boenninghausenia sessilicarpa	radially symmetrical flower	visible		floral guide mimics anther colour	2700
Asteraceae	Aster ageratoides cf	radially symmetrical inflorescence	hidden		inflorescence mimics androeceum	3400
Asteraceae	Aster brachytrichus	radially symmetrical inflorescence	hidden		inflorescence mimics androeceum	3900
Asteraceae	Aster jeffreyanus	radially symmetrical inflorescence	hidden			3900

Asteraceae	Aster oreophilus	radially symmetrical inflorescence	hidden		inflorescence mimics androeceum	3200
Asteraceae	Bidens pilosa var. radiata	radially symmetrical inflorescence	hidden			2700
Asteraceae	Carpesium scapiforme	radially symmetrical inflorescence	hidden			3200
Asteraceae	Cirsium lidiangense	radially symmetrical inflorescence	hidden			2700
Asteraceae	Cremanthodium campanulatum	radially symmetrical inflorescence	hidden			4100
Asteraceae	Cremanthodium nobile	radially symmetrical inflorescence	hidden			3900
Asteraceae	Eupatorium heterophyllum	radially symmetrical inflorescence	hidden			2700
Asteraceae	Galinsoga parviflora	radially symmetrical inflorescence	hidden		inflorescence mimics androeceum	3200
Asteraceae	Hippolytia delavayi	radially symmetrical inflorescence	hidden			4200
Asteraceae	Inula helianthus-aquatilis	radially symmetrical inflorescence	hidden		inflorescence mimics androeceum	2700
Asteraceae	Leontopodium andersonii	radially symmetrical inflorescence	hidden			3200
Asteraceae	Leontopodium calocephalum	radially symmetrical inflorescence	hidden			4100
Asteraceae	Leontopodium sinense	radially symmetrical inflorescence	hidden			2700
Asteraceae	Ligularia alatipes	radially symmetrical inflorescence	hidden		inflorescence mimics androeceum	2700
Asteraceae	Ligularia alatipes	radially symmetrical inflorescence	hidden			3400
Asteraceae	Ligularia cymbulifera	radially symmetrical inflorescence	hidden			3900
Asteraceae	Ligularia vellerea	radially symmetrical inflorescence	hidden		inflorescence mimics androeceum	3600
Asteraceae	Parasenecio lidjiangensis	radially symmetrical inflorescence	hidden		inflorescence mimics androeceum	3900
Asteraceae	Saussurea ciliaris	radially symmetrical inflorescence	hidden			4100
Asteraceae	Saussurea leontodontoides	radially symmetrical inflorescence	hidden			3900
Asteraceae	Senecio laetus	radially symmetrical inflorescence	hidden			2700
Asteraceae	Senecio spathiphyllus	radially symmetrical inflorescence	hidden		floral guide mimics anther colour	2700
Asteraceae	Soroseris hookeriana	radially symmetrical inflorescence	hidden		floral guide mimics anther colour	4100
Asteraceae	Syncalathium souliei	radially symmetrical inflorescence	hidden			4100

Asteraceae	Tagetes erecta	radially symmetrical inflorescence	hidden			2700
Asteraceae	Tagetes patula	radially symmetrical inflorescence	hidden			2700
Asteraceae	Taraxacum borealisinense	radially symmetrical inflorescence	hidden		inflorescence mimics androeceum	3400
Asteraceae	Wedelia urticifolia	radially symmetrical inflorescence	hidden			2200 (Yaxi)
Astraceae	Cremanthodium smithianum	radially symmetrical inflorescence	hidden			4200
Juncaceae	Juncus gracilicaulis	radially symmetrical inflorescence	no nectar			3200
Balsaminaceae	Impatiens amplexicaulis	zygomorphic flower	hidden			3200
Balsaminaceae	Impatiens delavayi	zygomorphic flower	hidden		floral guide mimics anther colour	3200
Balsaminaceae	Impatiens desmantha	zygomorphic flower	hidden		floral guide mimics anther colour	3200
Balsaminaceae	Impatiens margaritifera	zygomorphic flower	hidden		floral guide mimics anther colour	4100
Balsaminaceae	Impatiens radiata	zygomorphic flower	hidden		floral guide mimics anther colour	3200
Balsaminaceae	Impatiens uliginosa	zygomorphic flower	hidden		floral guide mimics anther colour	2700
Bignoniaceae	Incarvillea lutea	zygomorphic flower	hidden		floral guide mimics anther colour	3200
Bignoniaceae	Incarvillea mairei	zygomorphic flower	hidden		floral guide mimics anther colour	3200
Caprifoliaceae	Abelia dielsii	zygomorphic flower	hidden			3200
Caprifoliaceae	Dipelta yunnanensis	zygomorphic flower	hidden		floral guide mimics anther colour	3200
Fabaceae	Astragalus camptodontus	zygomorphic flower	hidden		hidden pollen	2700
Fabaceae	Astragalus pullus	zygomorphic flower	hidden		hidden pollen	3400
Fabaceae	Cheseneya polystichoides	zygomorphic flower	hidden		hidden pollen	3400
Fabaceae	Crotalaria yunnanensis	zygomorphic flower	hidden		hidden pollen	2700
Fabaceae	Glycyrrhiza yunnanensis	zygomorphic flower	hidden		hidden pollen	2700
Fabaceae	Hylodesmum williamsii	zygomorphic flower	hidden		hidden pollen	3200
Fabaceae	Indigofera cf	zygomorphic flower	hidden		hidden pollen	4100
Fabaceae	Indigofera pendula	zygomorphic flower	hidden		hidden pollen	3200
Fabaceae	Lespedeza formosa	zygomorphic flower	hidden		hidden pollen	3200
Fabaceae	Lespedeza forrestii	zygomorphic flower	hidden		hidden pollen	3200
Fabaceae	Lotus corniculatus	zygomorphic flower	hidden		hidden pollen	2700
Fabaceae	Medicago lupulina	zygomorphic flower	hidden		hidden pollen	3200
Fabaceae	Oxytropis yunnanensis	zygomorphic flower	hidden		hidden pollen	4100
Fabaceae	Parochetus communis	zygomorphic flower	hidden		hidden pollen	2700

Fabaceae	Tibetia yunnanensis	zygomorphic flower	hidden		floral guide mimics anther colour and hidden pollen	3200
Fabaceae	Trifolium repens cf	zygomorphic flower	hidden		hidden pollen	2700
Iridaceae	Iris tectorum	zygomorphic flower	hidden			3200
Lamiaceae	Ajuga forrestii	zygomorphic flower	hidden		hidden pollen	3200
Lamiaceae	Ajuga lupulina	zygomorphic flower	hidden		hidden pollen	3600
Lamiaceae	Clerodendrum yunnanense	zygomorphic flower	hidden		hidden pollen	2700
Lamiaceae	Clinopodium megalanthum	zygomorphic flower	hidden		hidden pollen	3200
Lamiaceae	Dracocephalum bullatum	zygomorphic flower	hidden		hidden pollen	4100
Lamiaceae	Eriophyton wallichii	zygomorphic flower	hidden		hidden pollen	4200
Lamiaceae	Galeopsis bifida	zygomorphic flower	hidden		floral guide mimics anther colour and hidden pollen	3200
Lamiaceae	Isodon irroratus	zygomorphic flower	hidden		hidden pollen	3200
Lamiaceae	Isodon phyllopodus	zygomorphic flower	hidden		hidden pollen	2700
Lamiaceae	Leonurus japonicus	zygomorphic flower	hidden		hidden pollen	2700
Lamiaceae	Nepeta wilsonii	zygomorphic flower	hidden		hidden pollen	3200
Lamiaceae	Phlomis atropurpurea	zygomorphic flower	hidden		hidden pollen	3200
Lamiaceae	Phlomis likiangensis	zygomorphic flower	hidden		hidden pollen	3400
Lamiaceae	Phlomis melanantha	zygomorphic flower	hidden		hidden pollen	3400
Lamiaceae	Prunella hispida	zygomorphic flower	hidden		hidden pollen	2700
Lamiaceae	Salvia aerea	zygomorphic flower	hidden		hidden pollen	3200
Lamiaceae	Salvia digitaloides	zygomorphic flower	hidden		hidden pollen	3400
Lamiaceae	Salvia flava	zygomorphic flower	hidden		floral guide mimics anther colour and hidden pollen	3600
Lamiaceae	Salvia trijuga	zygomorphic flower	hidden		hidden pollen	3200
Lamiaceae	Scutellaria amoena	zygomorphic flower	hidden		hidden pollen	3200
Lamiaceae	Scutellaria likiangensis	zygomorphic flower	hidden		hidden pollen	3200
Lamiaceae	Skapanthus oreophilus	zygomorphic flower	hidden		hidden pollen	3200
Lamiaceae	Stachys japonica Miq.	zygomorphic flower	hidden		hidden pollen	3200
Loranthantaceae	Taxillus colourea	zygomorphic flower	hidden			3200
Loranthantaceae	Taxillus delavayi	zygomorphic flower	hidden			3200
Orchidaceae	Gymnadenia conopsea	zygomorphic flower	hidden		hidden pollen	3600
Orchidaceae	Habenaria delavayi	zygomorphic flower	hidden		hidden pollen	2700

Orchidaceae	Neottia megalochila	zygomorphic flower	hidden		hidden pollen	3200 (Yunshanping)
Orchidaceae	Neottianthe calcicola	zygomorphic flower	hidden		hidden pollen	3600
Orchidaceae	Pecteilis susannae	zygomorphic flower	hidden		hidden pollen	2200 (Yaxi)
Orchidaceae	Satyrium ciliatum	zygomorphic flower	hidden		hidden pollen	3200
Orchidaceae	Spiranthes sinensis	zygomorphic flower	hidden		hidden pollen	3400
Orobanchaceae	Boschniakia himalaica	zygomorphic flower	hidden		hidden pollen	3200
Orobanchaceae	Pedicularis comptoniaefolia	zygomorphic flower	hidden		hidden pollen	3200
Orobanchaceae	Pedicularis crenata	zygomorphic flower	hidden		hidden pollen	2700
Orobanchaceae	Pedicularis densispica	zygomorphic flower	hidden		hidden pollen	3200
Orobanchaceae	Pedicularis likiangensis	zygomorphic flower	hidden		hidden pollen	4100
Orobanchaceae	Pedicularis lutescens	zygomorphic flower	hidden		hidden pollen	3200
Orobanchaceae	Pedicularis rex	zygomorphic flower	hidden		hidden pollen	2700
Orobanchaceae	Phtheirospermum tenuisectum	zygomorphic flower	hidden		floral guide mimics anther colour and hidden pollen	3200
Papaveraceae	Corydalis calcicola	zygomorphic flower	hidden		hidden pollen	3600
Papaveraceae	Corydalis delavayi	zygomorphic flower	hidden		floral guide mimics anther colour and hidden pollen	3600
Papaveraceae	Corydalis gracillima var. microcalcarata	zygomorphic flower	hidden		hidden pollen	3400
Papaveraceae	Corydalis pachycentra	zygomorphic flower	hidden		hidden pollen	3600
Papaveraceae	Corydalis smithiana	zygomorphic flower	hidden		hidden pollen	3200
Phrymaceae	Mimulus bodinieri	zygomorphic flower	hidden		floral guide mimics anther colour and hidden pollen	2700
Ranunculaceae	Aconitum stapfianum	zygomorphic flower	hidden			3200
Ranunculaceae	Delphinium beesianum	zygomorphic flower	hidden		hidden pollen	4100
Ranunculaceae	Delphinium grandiflorum	zygomorphic flower	hidden		hidden pollen	2700
Scrophulariaceae	Scrophularia spicata	zygomorphic flower	hidden			3200
Violaceae	Viola biflora var. rockiana	zygomorphic flower	hidden		floral guide mimics anther colour and hidden pollen	3900
Violaceae	Viola confertifolia	zygomorphic flower	hidden		floral guide mimics anther colour and hidden pollen	3600
Violaceae	Viola delavayi	zygomorphic flower	hidden		floral guide mimics anther colour and hidden pollen	2700
Violaceae	Viola philippica	zygomorphic flower	hidden		hidden pollen	2700

Zingiberaceae	Roscoea cautleoides	zygomorphic flower	hidden		floral guide mimics anther colour and hidden pollen	3200
Zingiberaceae	Roscoea schneideriana	zygomorphic flower	hidden		floral guide mimics anther colour and hidden pollen	2700
Zingiberaceae	Roscoea tibetica	zygomorphic flower	hidden		floral guide mimics anther colour and hidden pollen	3400
Araceae	Arisaema franchetianum	zygomorphic flower	hidden or no nectar			2700
Araceae	Arisaema erubescens	zygomorphic flower	hidden or no nectar			3200
Aristolochiaceae	Aristolochia faucimaculata	zygomorphic flower	hidden or no nectar			3200
Campanulaceae	Lobelia sessilifolia	zygomorphic flower	hidden or no nectar			2700
Orchidaceae	Galearis spathulata	zygomorphic flower	hidden or no nectar		hidden pollen	4100
Orobanchaceae	Pedicularis dunniana	zygomorphic flower	hidden or no nectar		hidden pollen	4100
Commelinaceae	Commelina maculata	zygomorphic flower	no nectar		staminodes mimic stamens	2700
Commelinaceae	Murdannia divergens	zygomorphic flower	no nectar		staminodes mimic stamens	2700
Commelinaceae	Streptolirion volubile	zygomorphic flower	no nectar		filmental hairs mimic pollen colour	3200
Orchidaceae	Amitostigma basifoliatum	zygomorphic flower	no nectar		hidden pollen	3200
Orchidaceae	Cypripedium flavum	zygomorphic flower	no nectar		hidden pollen	3200
Orchidaceae	Cypripedium plectrochilum	zygomorphic flower	no nectar		hidden pollen	3200
Orchidaceae	Cypripedium tibeticum	zygomorphic flower	no nectar		hidden pollen	3200
Orchidaceae	Orchis chusua	zygomorphic flower	no nectar		hidden pollen	3600
Orchidaceae	Pleione scopulorum	zygomorphic flower	no nectar		floral guide mimics anther colour and hidden pollen	3200
Orobanchaceae	Pedicularis alopecuros	zygomorphic flower	no nectar		hidden pollen	3200
Orobanchaceae	Pedicularis cephalantha	zygomorphic flower	no nectar		hidden pollen	3200
Orobanchaceae	Pedicularis elwesii	zygomorphic flower	no nectar		hidden pollen	3900
Orobanchaceae	Pedicularis gracilis	zygomorphic flower	no nectar		hidden pollen	3200
Orobanchaceae	Pedicularis gruina	zygomorphic flower	no nectar		hidden pollen	3200
Orobanchaceae	Pedicularis integrifolia	zygomorphic flower	no nectar		hidden pollen	3900
Orobanchaceae	Pedicularis lachnoglossa	zygomorphic flower	no nectar		hidden pollen	3900
Orobanchaceae	Pedicularis rhodotricha	zygomorphic flower	no nectar		hidden pollen	3900

Orobanchaceae	Pedicularis siphonantha	zygomorphic flower	no nectar		hidden pollen	4100
orobanchaceae	Pedicularis superba	zygomorphic flower	no nectar		hidden pollen	4100
Orobanchaceae	Pedicularis confertiflora	zygomorphic flower	no nectar		hidden pollen	4200
Ericaceae	Rhododendron fastigiatum	zygomorphic flower	potentially visible			3900
Scrophulariaceae	Verbascum thapsus	zygomorphic flower	potentially visible		filmental hairs mimic pollen colour	2700
Ericaceae	Rhododendron decorum	zygomorphic flower	visible		floral guide mimics anther colour	3200
Ericaceae	Rhododendron hippophaeoides cf	zygomorphic flower	visible			3200
Ericaceae	Rhododendron lepidotum	zygomorphic flower	visible			3200
Ericaceae	Rhododendron yunnanense	zygomorphic flower	visible		floral guide mimics anther colour	3200
Hydrangenaceae	Philadelphus calvescens	zygomorphic flower	visible			3200
Liliaceae	Lilium taliense	zygomorphic flower	visible			3200
Liliaceae	Lloydia tibetica	zygomorphic flower	visible		floral guide mimics anther colour	3400
Liliaceae	Lloydia yunnanensis	zygomorphic flower	visible			3200
Liliaceae	Notholirion bulbuliferum	zygomorphic flower	visible			3600
Nartheciaceae	Aletris pauciflora var. khasiana	zygomorphic flower	visible			3200
Orchidaceae	Peristylus coeloceras	zygomorphic flower	visible		hidden pollen	3400
Orchidaceae	Peristylus forceps	zygomorphic flower	visible		hidden pollen	3200
Plantaginaceae	Veronica piroliformis	zygomorphic flower	visible			3200
Rosaceae	Agrimonia pilosa var. nepalensis	zygomorphic flower	visible			2700
Rosaceae	Fragaria vesca	zygomorphic flower	visible			2700
Rosaceae	Geum aleppicum	zygomorphic flower	visible		stigma mimics stamen	2700
Rosaceae	Rosa sericea	zygomorphic flower	visible		stigma mimics stamen	2700
Rosaceae	Rubus biflorus cf	zygomorphic flower	visible			2700
Rosaceae	Rubus fockeanus	zygomorphic flower	visible			3600
Rosaceae	Sanguisorba filiformis	zygomorphic flower	visible			2700
Rosaceae	Sanguisorba officinalis	zygomorphic flower	visible			2700
Rosaceae	Spiraea lichiangensis	zygomorphic flower	visible			greenhouse

Table S2. Summary of the results shown in Table S1 indication the number and percentage of species with features relevant in the context of nectar mimicry

Category	No. of species	Percentage (%)
species with radially symmetrical flowers	174	56.3
with glossy area	38	12.3
without glossy area	136	44.0
species with nectar visible/potentially visible	88	28.5
with glossy area	31	10.0
without glossy area	57	18.4
species with pollen-mimicking structure	75	24.3
species with glossy and with pollen-mimicking structure	17	5.5
species with hidden or no nectar	221	71.5
species with zygomorphic flowers	135	43.7
with glossy area	1	0.3
all species	309	100.0

Table S3. RGB-values of flowers in China from false colour photos as calculated in Irvan View (in bracts bee-visible range of wavelength)
* indicates glossy area, adjacent area was used for calculations; Colour refers to the human-visible colour; (UV) refers to the ultraviolet reflecting (+) or UV-absorbing (-) properties

Species	Familiy	Structure	Colour (UV)	red (green)	green (blue)	blue (UV)
Parnassia wightiana	Celastraceae	staminode *	green (-)	66.82	10.38	168.07
		staminode, adjacent area	green (-)	63.89	0.32	31.83
		petal, middle	white (-)	117.89	89.93	24.47
Saxifraga unguiculata	Saxifragaceae	protuberance, petal *	yellow (-)	113.15	0.86	208.64
		petal, adjacent area	yellow (-)	115.25	0.16	69.21
		petal, middle	yellow (-)	127.71	0.26	70.65
Saxifraga nigrograndulosa	Saxifragaceae	protuberance, petal *	yellow (-)	243.3	11.27	240.73
		protuberance, adjacent area	yellow (-)	129.71	0.21	86.37
		petal, middle	yellow (-)	151.33	0.23	92.3
Saxifraga diversifolia	Saxifragaceae	protuberance on petal *	yellow (-)	108.55	0.52	200.59
		protuberance, adjacent area	yellow (-)	104.57	0.29	73.39
		petal, middle	yellow (-)	123.66	0.31	69.98
		nectary	yellow (-)	103.7	40.04	203.43
Saxifraga melanocentra	Saxifragaceae	disc *	black (-)	98.02	130.95	211.37
		margin of disc	black (-)	34.63	39.4	48.94
		petal, middle	white (-)	247.96	252.33	30.53
Codonopsis graminifolia	Campanulaceae	basis of petal *	black (-)	93.57	110.16	212.5
		basis of petal, adjacent area	black (-)	6.96	13.63	40.56
		petal, middle	blue (+)	87.41	173.05	234.38
Saxifraga signata		protuberance on petal *	yellow (-)	231.82	140.64	230.61
		petal, middle	yellow (-)	180.8	54.13	82.46
Anaphalis nepalensis	Asteraceae	petal, base *	white (-)	159.48	160.61	254.41
		petal, tip	white (-)	96.17	77.72	82.48

Trollius yunnanensis	Ranunculaceae	staminode *		137.84	18.08	228.76
		staminode, adjacent area		107.53	0	109.38
Parnassia delavayi	Celeastraceae	staminode *	green (-)	18.54	0.96	242.94
		staminode, adjacent area	green (-)	14.84	0.22	72.98
		petal middle	white (-)	127.07	137.06	178.17
Parnassia yunnanensis	Celeastraceae	staminode *	green (-)	53.06	30.06	198.79
		staminode, adjacent area	green (-)	23.83	0.01	26.66
		petal middle	green (-)	141.58	70.26	173.4
Potentilla lancinata cf	Rosaceae	protuberance *	yellow (-)	52.05	2.21	228.28
		protuberance, adjacent area	yellow (-)	51.02	0.68	35.42
		petal, base	yellow (-)	78.97	0.29	29.49

Table S4. RGB-values of flowers in Germany, Switzerland and Australia from false colour photos in Irvan View (in bracts bee-visible range of wavelength)
* indicates glossy area, adjacent area was used for calculations; Colour refers to the human-visible colour; (UV) refers to the ultraviolet reflecting (+) or UV-absorbing (-) properties

Species	Familiy	Structure	Colour (UV)	red (green)	green (blue)	blue (UV)
Saxifraga stolonifera	Saxifragaceae	disc *	yellow (-)	111.45	0.65	247.99
		disc, adjacent area	yellow(-)	101.4	0.55	69.23
		petal, downward	white (-)	170.36	174.96	239.7
Rudbeckia fuliginosa	Asteraceae	anthers *	black (-)	85.98	93.63	238.26
		anthers, adjacent area	black (-)	16.89	7.88	58.79
		petal, base	yellow (-)	243.69	5.13	107.07
Tulipa gesneriana	Liliaceae	petal, base *	black (-)	119.02	126.78	176.57
		petal, base adjacent	black (-)	30.11	35.24	54.42
		petal middle	red (-)	27.85	30.28	45.2
Massonia pustulata	Asparagaceae	nectar *	black (-)	38.52	28.96	139.54
		nectar, adjacent	black (-)	41.34	32.01	19.29
		filament	white +	181.17	177.33	10.27
Parnassia palustris	Celastraceae	staminode, head *	yellow (-)	92.93	30.4	142.8
		staminode, adjacent area	yellow (-)	57.5	11.58	14
		petal		134.05	119.79	21.48
Solanum dulcamara	Solanaceae	floral guide *	green (-)	163.88	152.16	133.02
		floral guide, adjacent area	green (-)	142.2	89.99	36.35
		petal	pink +	112.43	143.69	66.83
Anemone coronaria	Ranunculaceae	petal, base *	white (-)	216.83	225.38	104.44
		petal, base, adjacent	white (-)	182.17	191.58	18.22
		petal, middle	red (-)	41.73	0.38	14.23
Swainsonia formosa	Fabaceae	bulb *	black (-)	230.75	241.51	218.32
		bulb, adjacent	black (-)	12.72	24.61	24.83
		corolla	red (-)	17.55	20.37	8.3

Table S5. Species with glossy structures in the Hengduan mountains. Species, from which quantitative measurements were taken are highlighted in yellow. Remarks explain the reasons why from some species were excluded.

**SPECIES (FAMILY)**	**VISIBLE NECTAR & GLOSSY STRUCTURE**	**REMARKS**	**COLOUR PHOTO / UV-PHOTO / FALSE COLOUR PHOTO**
Anaphalis chlamydophylla (Asteraceae)	glossy floral guide with hidden nectar	photos taken without sunshine, inflorescences only open in full sunshine, evidence for glossy structures from personal observation	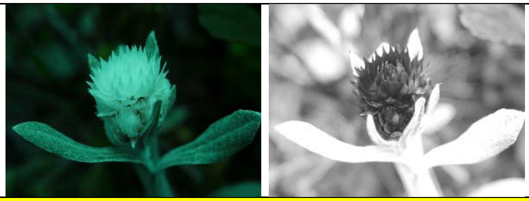
Anaphalis nepalensis (Asteracee)	glossy floral guide with hidden nectar	quantitative data collected	Photos see Fig. 1g-i
Anaphalis yunnanensis cf (Asteraceae)	glossy floral guide with hidden nectar	photos taken with a different camera system, photos of insufficient quality, evidence for glossy structures from personal observation	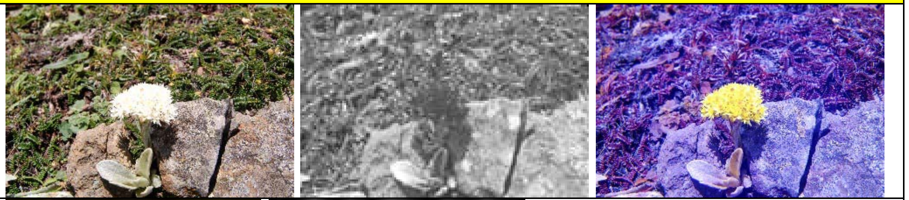
Codonopsis bulleyana (Campanulaceae)	radially symmetrical flower with visible nectar, glossy basal part of petal	photos taken without sunshine, evidence for glossy structures from personal observation	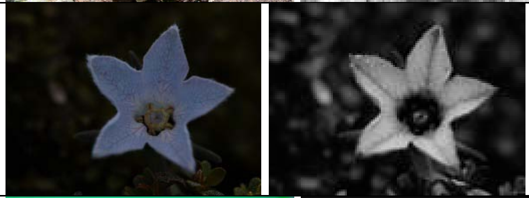
Codonopsis convolvulacea (Campanulaceae)	radially symmetrical flower with visible nectar, glossy basal part of petal	photos taken without sunshine and different camera system	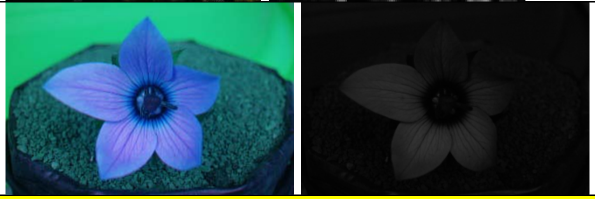
Codonopsis graminifolia (Campanulaceae)	radially symmetrical flower with visible nectar, glossy basal part of petal	quantitative data collected	Photos see Fig. 1m-o
Cyananthus formosus (Campanulaceae)	glossy part of petal with hidden nectar	photos taken without sunshine and different camera system, evidence for glossy structures from personal observation	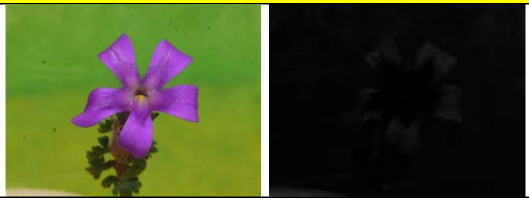

Cyananthus longiflorus (Campanulaceae)	glossy part of petal with hidden nectar	photos taken without sunshine and different camera system, evidence for glossy structures from personal observation	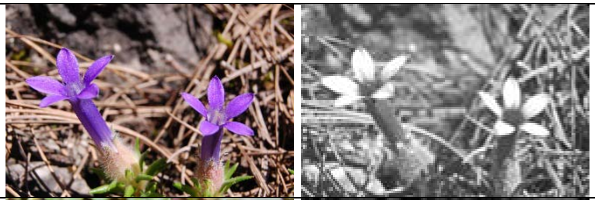
Cyananthus hookeri (Campanulaceae)	glossy part of petal with hidden nectar	photos taken without sunshine and different camera system, evidence for glossy structures from personal observation	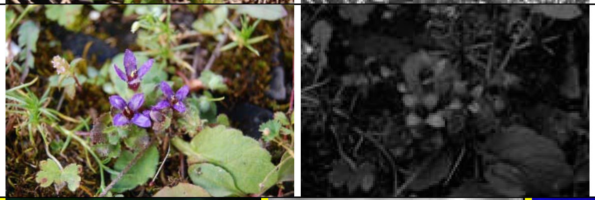
Parnassia delavayi (Celastraceae)	glossy staminodes, nectar visible	quantitative data collected	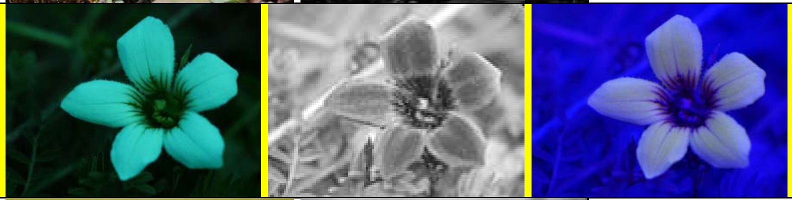
Parnassia mysorensis (Celastraceae)	glossy staminodes, visible	photos taken without sunshine and different camera system, evidence for glossy structures from personal observation	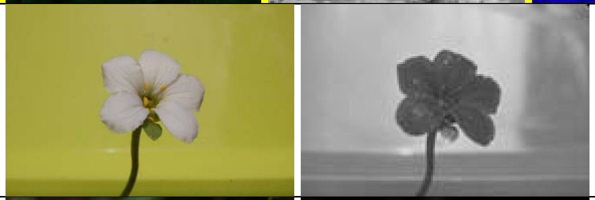
Parnassia tenella (Celastraceae)	glossy staminodes, nectar visible	photos taken without sunshine and different camera system, evidence for glossy structures from personal observation	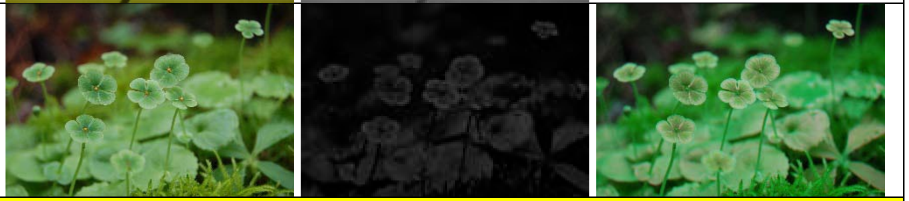
Parnassia wightiana (Celastraceae)	glossy staminodes, nectar visible	quantitative data collected	Photos see Fig. 1j-l
Parnassia yunnanensis (Celastraceae)	glossy staminodes, nectar visible	quantitative data collected	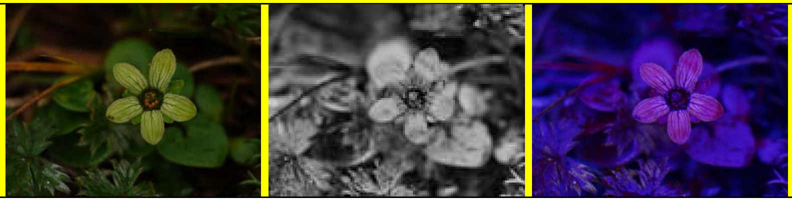

Geranium delavayi (Erodiaceae)	radially symmetrical flower with visible nectar, glossy basal part of petals and sepals	photos taken without sunshine, evidence for glossy structures on petal and sepals from personal observation	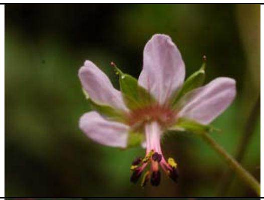 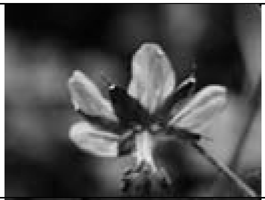
Geranium strictipes (Erodiaceae)	radially symmetrical flower with visible nectar, glossy basal part of petals and sepals	photos taken without sunshine, evidence for glossy structures on petals and sepals from personal observation	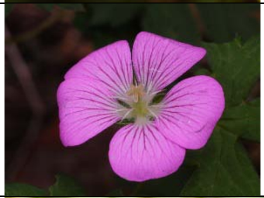 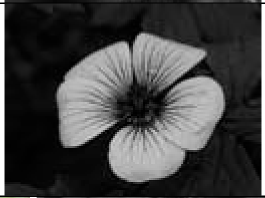
Swertia cincta (Gentianaceae)	radially symmetrical flower with visible nectar, glossy basal part of petal	photos taken without sunshine and different camera system, evidence for glossy structures from personal observation	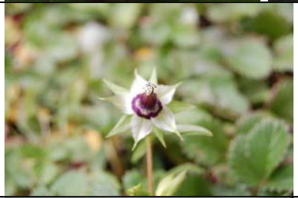 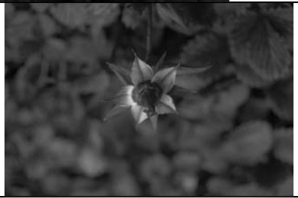
Swertia macrosperma (Gentianaceae)	radially symmetrical flower with visible nectar, glossy basal part of petal	photos taken without sunshine and different camera system, evidence for glossy structures from personal observation,	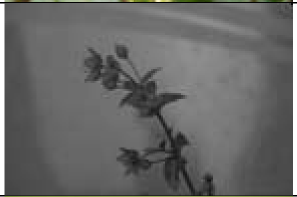 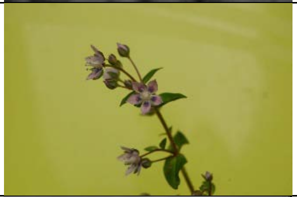
Swertia punicea (Gentianaceae)	radially symmetrical flower with visible nectar, glossy basal part of petal	photos taken without sunshine and different camera system, evidence for glossy structures from personal observation	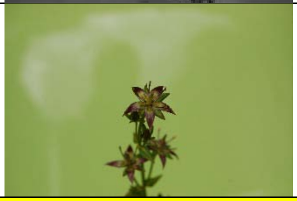 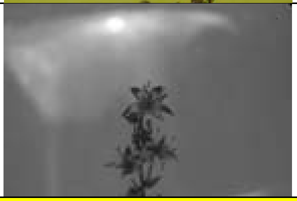
Trollius yunnanensis (Ranunculaceae)	glossy staminodes, no nectar	quantitative data collected	Photos see Fig. 1p-r
Potentilla anserina (Rosaceae)	radially symmetrical flower with visible nectar, glossy central structures and glossy sepals	photos taken ithout sunshine, evidence for glossy structures in center and on sepals from personal observation	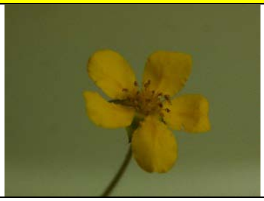 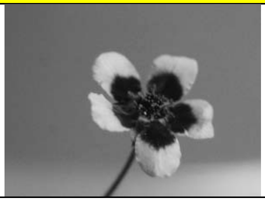

Potentilla articulata (Rosaceae)	radially symmetrical flower with visible nectar, glossy central structures and glossy sepals	photos taken with different camera system, evidence for glossy structures in center and on sepals from personal observation	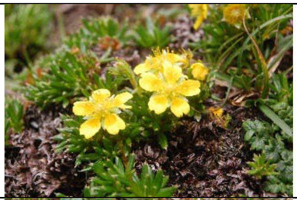 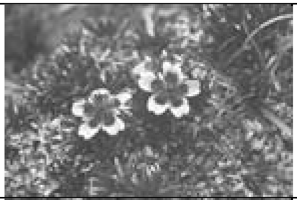
Potentilla coriandrifolia (Rosaceae)	radially symmetrical flower with visible nectar, glossy central structures and glossy sepals	photos taken without sunshine and different camera system, evidence for glossy structures in center and on sepals in center and on sepals from personal observation	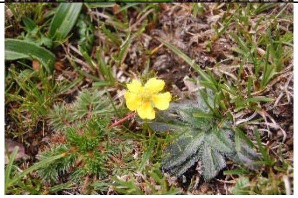 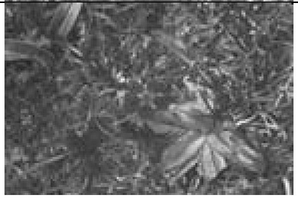
Potentilla eriocarpa (Rosaceae)	radially symmetrical flower with visible nectar, glossy central structures and glossy sepals	photos of insufficient quality, evidence for glossy structures in center and on sepals from personal observation	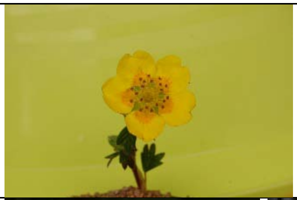 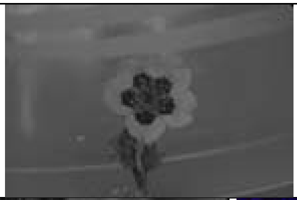 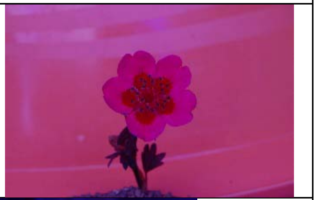
Potentilla fruticosa (Rosaceae)	radially symmetrical flower with visible nectar, glossy central structures and glossy sepals	photos taken with different camera system, evidence for glossy structures in center and on sepals from personal observation	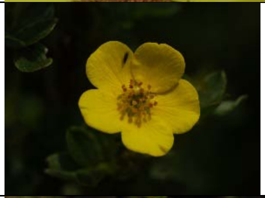 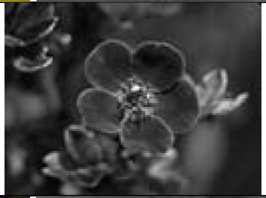 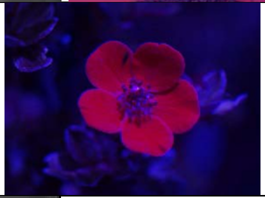
Potentilla glabra (Rosaceae)	radially symmetrical flower with visible nectar, glossy central structures and glossy sepals	photos taken with different camera system, evidence for glossy structures in center and on sepals from personal observation	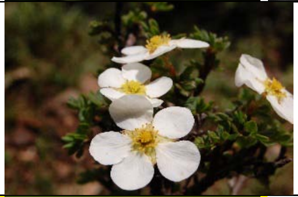 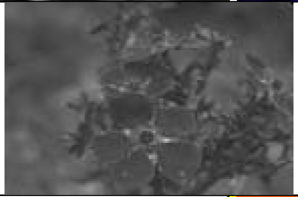
Potentilla lancinata cf (Rosaceae)	radially symmetrical flower with visible nectar, glossy central structures and glossy sepals	quantitative data collected	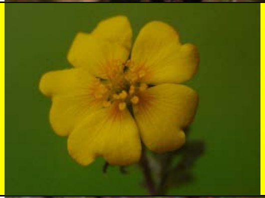 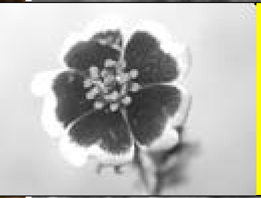 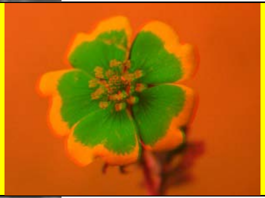
Potentilla peduncularis var. curta (Rosaceae)	radially symmetrical flower with visible nectar, glossy central structures and glossy sepals	photos taken without sunshine and different camera system, evidence for glossy structures from personal observation	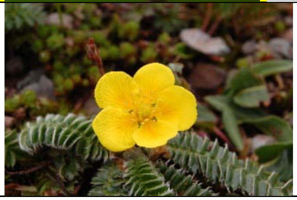 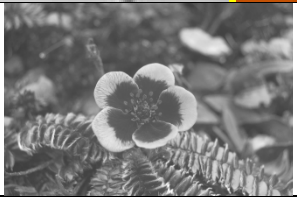

Saxifraga melanocentra (Saxifragaceae)	radially symmetrical flower with visible nectar, glossy disc	quantitative data collected	Photos see Fig. 1a-c
Saxifraga rufescens (Saxifragaceae)	zygomorphic flower with visible nectar, glossy disc	photos taken without sunshine, evidence for glossy structures from personal observation	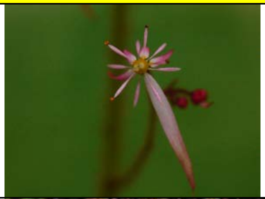 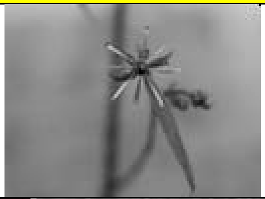
Saxifraga aristulata (Saxifragaceae)	glossy protuberances, nectar visible	photos taken without sunshine and different camera system, evidence for glossy structures from personal observation	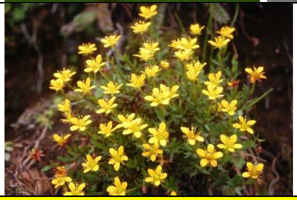 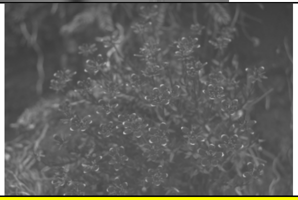
Saxifraga diversifolia, (Saxifragaceae)	glossy protuberances, nectar visible	quantitative data collected	Photos see Fig. 2d-f
Saxifraga filicaulis (Saxifragaceae)	glossy protuberances, nectar visible	photos taken without sunshine and different camera system, evidence for glossy structures from personal observation	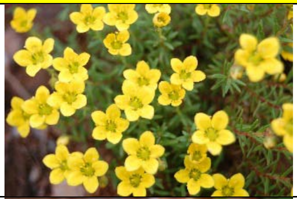 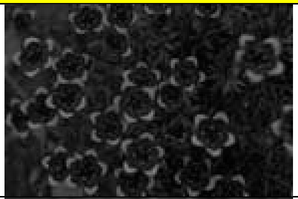
Saxifraga gemmipara (Saxifragaceae)	glossy protuberances, nectar visible	photos taken without sunshine and different camera system, evidence for glossy structures from personal observation	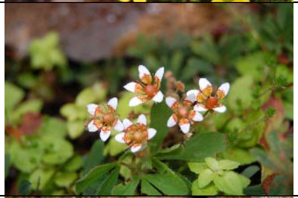 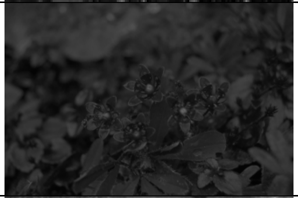
Saxifraga hispidula (Saxifragaceae)	glossy protuberances, nectar visible	photos taken without sunshine and different camera system, evidence for glossy structures from personal observation	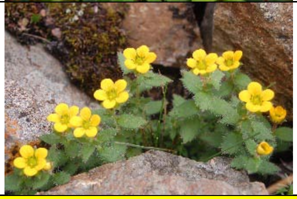 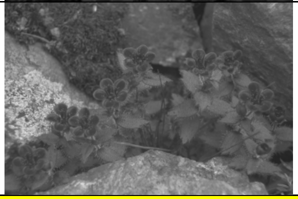
Saxifraga nigroglandulosa (Saxifragaceae)	glossy protuberances, nectar visible	quantitative data collected	Photos see Fig. 2g-i

Saxifraga strigosa (Saxifragaceae)	glossy protuberances, nectar visible	photos taken without sunshine and different camera system, evidence for glossy structures from personal observation	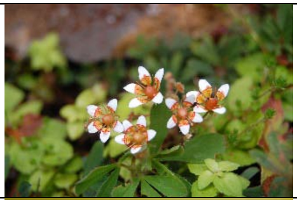 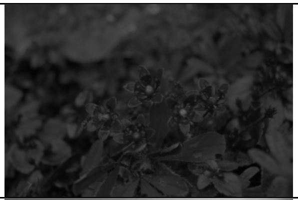
Saxifraga wallichiana (Saxifragaceae)	glossy protuberances, nectar visible	photos taken without sunshine and different camera system, evidence for glossy structures from personal observation	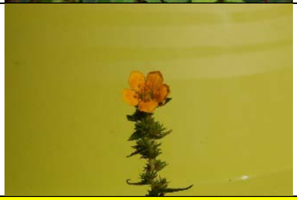 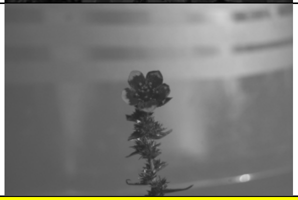
Saxifraga signata (Saxifragaceae)	glossy protuberances, nectar visible	quantitative data collected	Photos see Fig. 1d-f
Saxifraga unguiculata (Saxifragaceae)	glossy protuberances, nectar visible	quantitative data collected	Photos see Fig. 2a-c
Nicandra physalodes (Solanaceae)	radially symmetrical flower with no nectar	photos taken without sunshine and different camera system, evidence for glossy structures from personal observation	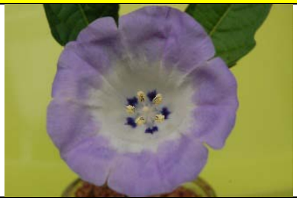 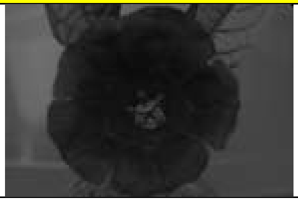 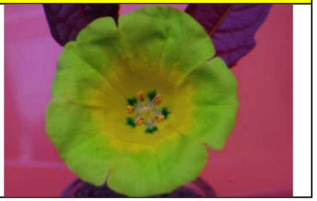

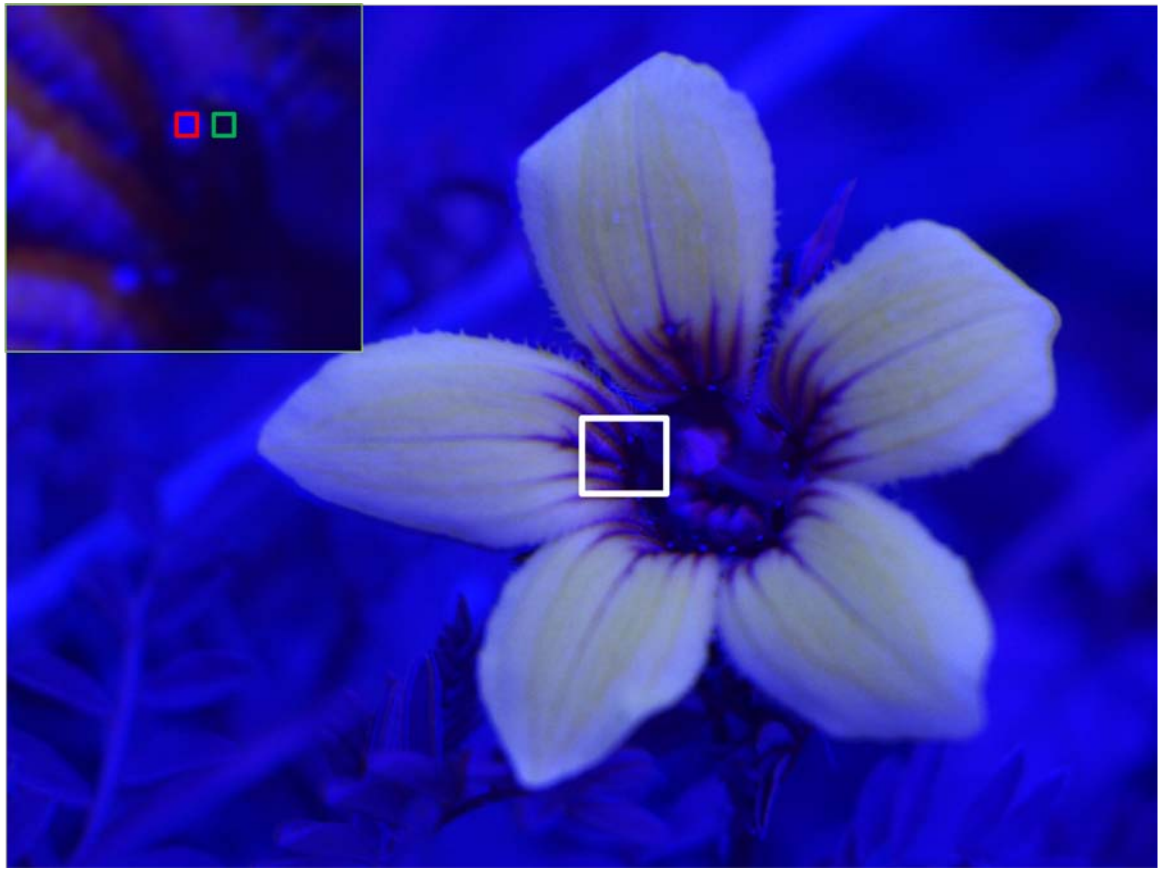

Fig. S1. Example of *Parnassia delavayi* for quantification of the difference in the reflection in the ultraviolet, blue, and green ranges of wavelengths was quantified using IrfanView image's histogram (free from www.irfanview.com). Enlarged area of the false color picture used for the measurements (white). Inset: Uniformly coloured, glossy area (red) and adjacent, non-glossy area of the same structure (green) were measured in the same way.

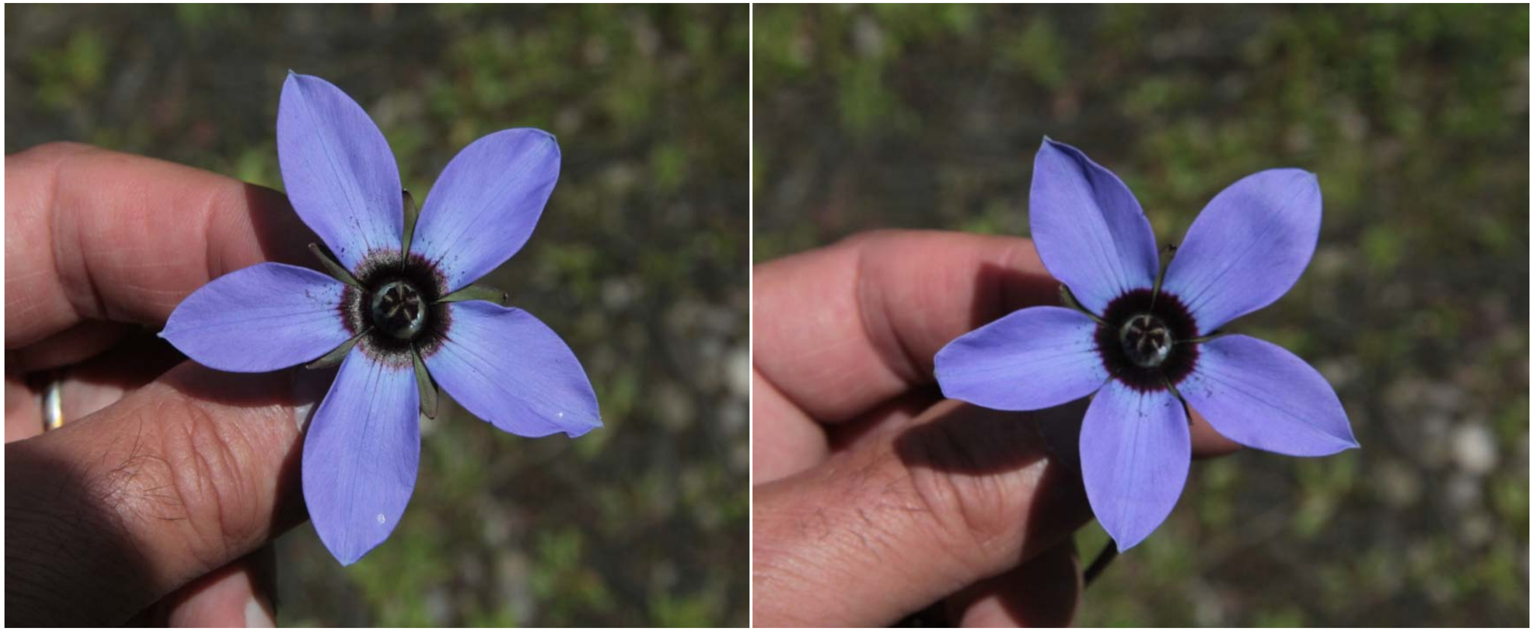

Fig. S2. Example of *Codonopsis graminifolia* showing that the gloss of the concave structures in the dark basal part of the petals is strictly dependent of the angle of the flower and the sun. Note the shadow on the fingers in order to estimate the position in relation to the sun.
